# Knockdown of *Foxg1* in supporting cells increases the trans-differentiation of supporting cells into hair cells in the neonatal mouse cochlea

**DOI:** 10.1007/s00018-019-03291-2

**Published:** 2019-09-04

**Authors:** Shasha Zhang, Yuan Zhang, Ying Dong, Lingna Guo, Zhong Zhang, Buwei Shao, Jieyu Qi, Han Zhou, Weijie Zhu, Xiaoqian Yan, Guodong Hong, Liyan Zhang, Xiaoli Zhang, Mingliang Tang, Chunjie Zhao, Xia Gao, Renjie Chai

**Affiliations:** 1 Key Laboratory for Developmental Genes and Human Disease, Ministry of Education, Institute of Life Sciences, Southeast University, Nanjing, 210096 China; 2 Co-Innovation Center of Neuroregeneration, Nantong University, Nantong, 226001 China; 3 Institute for Stem Cell and Regeneration, Chinese Academy of Science, Beijing, China; 4 Jiangsu Province High-Tech Key Laboratory for Bio-Medical Research, Southeast University, Nanjing, 211189 China; 5 Jiangsu Provincial Key Medical Discipline (Laboratory), Department of Otolaryngology Head and Neck Surgery, Affiliated Drum Tower Hospital of Nanjing University Medical School, Nanjing, 210008 China; 6 Key Laboratory of Hearing Medicine of NHFPC, ENT Institute and Otorhinolaryngology Department of Affiliated Eye and ENT Hospital, Shanghai Engineering Research Centre of Cochlear Implant, State Key Laboratory of Medical Neurobiology, Fudan University, Shanghai, 200031 China

**Keywords:** Foxg1, Hair cells, Supporting cells, Progenitors, Proliferation, Trans-differentiation

## Abstract

*Foxg1* is one of the forkhead box genes that are involved in morphogenesis, cell fate determination, and proliferation, and Foxg1 was previously reported to be required for morphogenesis of the mammalian inner ear. However, *Foxg1* knock-out mice die at birth, and thus the role of Foxg1 in regulating hair cell (HC) regeneration after birth remains unclear. Here we used Sox2^CreER/+^ Foxg1^loxp/loxp^ mice and Lgr5-EGFP^CreER/+^ Foxg1^loxp/loxp^ mice to conditionally knock down *Foxg1* specifically in Sox2+ SCs and Lgr5+ progenitors, respectively, in neonatal mice. We found that *Foxg1* conditional knockdown (cKD) in Sox2+ SCs and Lgr5+ progenitors at postnatal day (P)1 both led to large numbers of extra HCs, especially extra inner HCs (IHCs) at P7, and these extra IHCs with normal hair bundles and synapses could survive at least to P30. The EdU assay failed to detect any EdU+ SCs, while the SC number was significantly decreased in *Foxg1* cKD mice, and lineage tracing data showed that much more tdTomato+ HCs originated from Sox2+ SCs in *Foxg1* cKD mice compared to the control mice. Moreover, the sphere-forming assay showed that *Foxg1* cKD in Lgr5+ progenitors did not significantly change their sphere-forming ability. All these results suggest that *Foxg1* cKD promotes HC regeneration and leads to large numbers of extra HCs probably by inducing direct trans-differentiation of SCs and progenitors to HCs. Real-time qPCR showed that cell cycle and Notch signaling pathways were significantly down-regulated in *Foxg1* cKD mice cochlear SCs. Together, this study provides new evidence for the role of Foxg1 in regulating HC regeneration from SCs and progenitors in the neonatal mouse cochlea.

**Electronic supplementary material:**

The online version of this article (10.1007/s00018-019-03291-2) contains supplementary material, which is available to authorized users.

## Introduction

The loss of hair cells (HCs) is the main cause of sensorineural hearing loss, which is one of the most common health problems around the world. HC loss is irreversible in adult mammals, whereas HCs can be regenerated from supporting cells (SCs) in the inner ear of birds and fish [1]. Recent studies have shown that in newborn mice, HCs can also be regenerated from SCs, especially from a subset of Lgr5+ progenitor cells [2–8]. However, this regenerative ability is quickly lost as the mice age [2–4, 9, 10].

Recent studies have shown that several signaling pathways play important roles in HC regeneration by inducing the proliferation and differentiation of SCs and Lg5+ progenitors. The up-regulation of canonical Wnt signaling induces the proliferation of sensory precursors in the postnatal mouse cochlea [3, 4, 11–17], while Notch inhibition induces mitotic generation of HCs in the mammalian cochlea via activation of the Wnt pathway [12, 14, 18–25]. Also, their effect on differentiation and the generation of HCs is related to important genes such as *Atoh1* and *Neurog1* [26–34]. *Foxg1* (formerly called *Bf*-*1*) is one of the forkhead box (FOX) family genes, and it plays an important role in neuron development and has been reported to engage in crosstalk with Wnt, Notch, and TGFβ signaling in the brain and eye [35–43]. In the inner ear, *Foxg1* is expressed in almost all cell types in the cochlea, saccule, utricle, and canal cristae, and *Foxg1*-null mice have both morphological and histological defects in inner ear development, including shortened cochleae with multiple rows of HCs and the loss of crista neurons and horizontal ampulla [44, 45]. In addition, *Foxg1*-null mice demonstrate striking abnormalities in cochlear and vestibular innervation, including loss of all crista neurons and numerous fibers that overshoot the organ of Corti [45], and similar phenotypes have also been demonstrated for *Neurod1* mutations [33, 34, 46]. However, due to the postnatal lethality of *Foxg1*-null mice, the roles of Foxg1 in HC regeneration in the postnatal mouse cochlea have remained unknown.

The genes of the FOX family belong to an evolutionarily conserved family of transcription factors that contain a winged-helix DNA-binding domain. These genes play important roles in development, organogenesis, and carcinogenesis [47–51]. *Foxg1*, one member of the FoxG subfamily, is involved in human Rett syndrome, which presents with severe neural developmental problems, cognitive impairment, and growth retardation [52, 53]. In mouse embryos, *Foxg1* is expressed in the telencephalon, eye, foregut, and otic placode [54–56]. *Foxg1* knock-out mice, which die at the perinatal period, show hypoplasia of the telencephalon and abnormal eye and ear development [45, 55, 57]. In forebrain development, Foxg1 maintains the progenitor pools and inhibits neuronal differentiation, and it is down-regulated when progenitors undergo neuronal differentiation [55, 58–64]. In postnatal mice, Foxg1 also plays an important role in maintaining the hippocampal dentate gyrus progenitor pool, and the lack of Foxg1 promotes both gliogenesis and neurogenesis [24]. In the eye, Foxg1 is essential for the projection of retinal ganglion cells, closure of the optic fissure, and the formation of ciliary margin tissue [35, 36, 56, 65–69].

Being aware of the proliferation induction and differentiation repression of neuron progenitors by Foxg1 and the multiple rows of HCs in *Foxg1*-null mice, we hypothesized that Foxg1 might also regulate the proliferation and differentiation ability of inner ear SCs which include the HC progenitors, and are able to regenerate HCs in the postnatal mouse cochlea. Here we crossed Sox2-CreER mice and Lgr5-EGFP-CreERT2 mice with Foxg1-floxp mice to conditionally knockdown *Foxg1* in Sox2+ SCs and Lgr5+ progenitors, respectively, and then evaluated the proliferation and differentiation ability of the SCs and Lgr5+ progenitors. Our data suggest that *Foxg1* cKD in cochlear SCs and progenitors probably promote the direct trans-differentiation of SCs and Lgr5+ progenitors into HCs, but it does not significantly change the proliferation ability of SCs and Lgr5+ progenitors in neonatal mouse cochlea.

## Materials and methods

### Animals

Lgr5-EGFP-IRES-CreERT2 mice (Stock #008875, Jackson Laboratory) [4, 70, 71], Sox2-CreER mice (Stock #017593, Jackson Laboratory) [14], and Rosa26-tdTomato reporter mice (Stock #007914, Jackson Laboratory) [4, 72] of both sexes were used in the experiments. The mouse breeding strategy is shown in Fig. S1. The Foxg1-floxp mice were a gift from Prof. Chunjie Zhao from Southeast University [24]. Sox9-IRES-CreER mice were a gift from Prof. Fengchao Wang from the National Institute of Biological Sciences (NIBS), Beijing [73]. We performed all animal procedures according to protocols that were approved by the Animal Care and Use Committee of Southeast University and that were consistent with the National Institute of Health’s Guide for the Care and Use of Laboratory Animals. We made all efforts to minimize the number of animals used and to prevent their suffering.

### Genotyping PCR

Transgenic mice were genotyped using genomic DNA, which was extracted from mice tail tips by adding 180 µl 50 mM NaOH, incubating at 98 °C for 1 h, and then adding 20 µl 1 M Tris–HCl pH 7.0. The genotyping primers were used as follows: *Lgr5*: (F) CTG CTC TCT GCT CCC AGT CT; wild type (R) 5′-ATA CCC CAT CCC TTT TGA GC-3′; mutant (R) 5′-GAA CTT CAG GGT CAG CTT GC-3′. tdTomato: wild type (F) 5′-AAG GGA GCT GCA GTG GAG T-3′; wild type (R) 5′-CCG AAA ATC TGT GGG AAG TC-3′; mutant (F) 5′-GGC ATT AAA GCA GCG TAT C-3′; mutant (R) 5′-CTG TTC CTG TAC GGC ATG G-3′. *Sox2*: wild type (F) 5′-CTA GGC CAC AGA ATT GAA AGA TCT-3′; wild type (R) 5′-GTA GGT GGA AAT TCTA GCA TCA TCC-3′; mutant (F) 5′-GCG GTC TGG CAG TAA AAA CTA TC-3′; mutant (R) 5′-GTG AAA CAG CAT TGC TGT CAC TT-3′. *Foxg1*: wild type (F) 5′-ATA AAG ATT TGC TGA GTT GGA-3′; mutant (F) 5′-GCA TCG CAT TGT CTG AGT AGG TG-3′; (R) 5′-TGG AGG GGG AGA TAG GGC TAT-3′. *Sox9*: (F) 5′-GCC TGC ATT ACC GGT CGA TGC-3′; (R) 5′-CAG GGT GTT ATA AGC AAT CCC C-3′. The extracted genomic DNA and primers were used in the following PCR system to genotype the mice: genomic DNA 3 µl, primer mix 2 µl, 2 × PCR mix (Thermo) 10 µl, and H_2_O were added to a total volume of 20 µl. PCR conditions were an initial denaturing step of 3 min at 95 °C followed by 38 cycles of 30 s denaturation at 95 °C, 30 s annealing at 60 °C, and 30 s extension at 72 °C.

### In vivo cKD of *Foxg1* in Sox2+ SCs, Sox9+ SCs, and Lgr5+ progenitors in the mouse cochlea

Sox2^CreER/+^ Foxg1^loxp/loxp^ mice, Sox9^CreER/+^ Foxg1^loxp/loxp^ mice, and Lgr5-EGFP^CreER/+^ Foxg1^loxp/loxp^ mice were bred to conditionally knockdown *Foxg1* in Sox2+ SCs, Sox9+ SCs, and Lgr5+ progenitors, respectively. To activate the Cre enzyme, postnatal day (P)1 or P3 mice were injected with tamoxifen (Sigma) intraperitoneally (I.P.) (1.5 mg/25 g body weight for Sox2-CreER mice, 3.5 mg/25 g body weight for Lgr5-CreER mice, and 2 mg/25 g body weight for Sox9-CreER mice, which were all consistent with previous reports [4, 73, 74]). For each experiment, the control mice were also injected with the same amount of tamoxifen. Mice were sacrificed at different time points, and the cochleae were examined.

### Auditory brainstem response (ABR) test

P30 mice were I.P. injected with 0.01 g/ml pentobarbital sodium (100 mg/kg body weight) to achieve deep anesthesia, and a TDT System III workstation running SigGen32 software (Tucker-Davis Technologies) was used to test mice for closed-field ABR thresholds as previously described [75]. The ABR test was performed in a soundproof room, and three fine needle electrodes were inserted in the mice at the cranial vertex, underneath the tested ear, and at the back near the tail. ABR tone pips of 4 kHz, 8 kHz, 12 kHz, 16 kHz, 24 kHz, and 32 kHz were generated. Auditory thresholds were determined by decreasing the sound intensities from 90 dB in 10 dB steps until the lowest sound intensity at which the first wave could be recognized. The ABR data were analyzed using GraphPad Prism 6 software.

### In vivo lineage tracing of Sox2+ SCs in the cochlea

Sox2^CreER/+^ Foxg1^loxp/loxp^ mice were crossed with Foxg1^loxp/loxp^ Rosa26-tdTomato mice to get Sox2^CreER/+^ Foxg1^loxp/loxp^ Rosa26-tdTomato triple-positive mice and to lineage trace Sox2+ SCs in the cochleae. To activate the Cre enzyme, Sox2^CreER/+^ Foxg1^loxp/loxp^ Rosa26-tdTomato triple-positive mice were I.P. injected with tamoxifen at P3. Mice were sacrificed at P9, and the cochleae were examined. Sox2^CreER/+^ Rosa26-tdTomato mice were used as controls, and were injected with the same dose of tamoxifen.

### Immunostaining and image acquisition

**Fig. 1:**
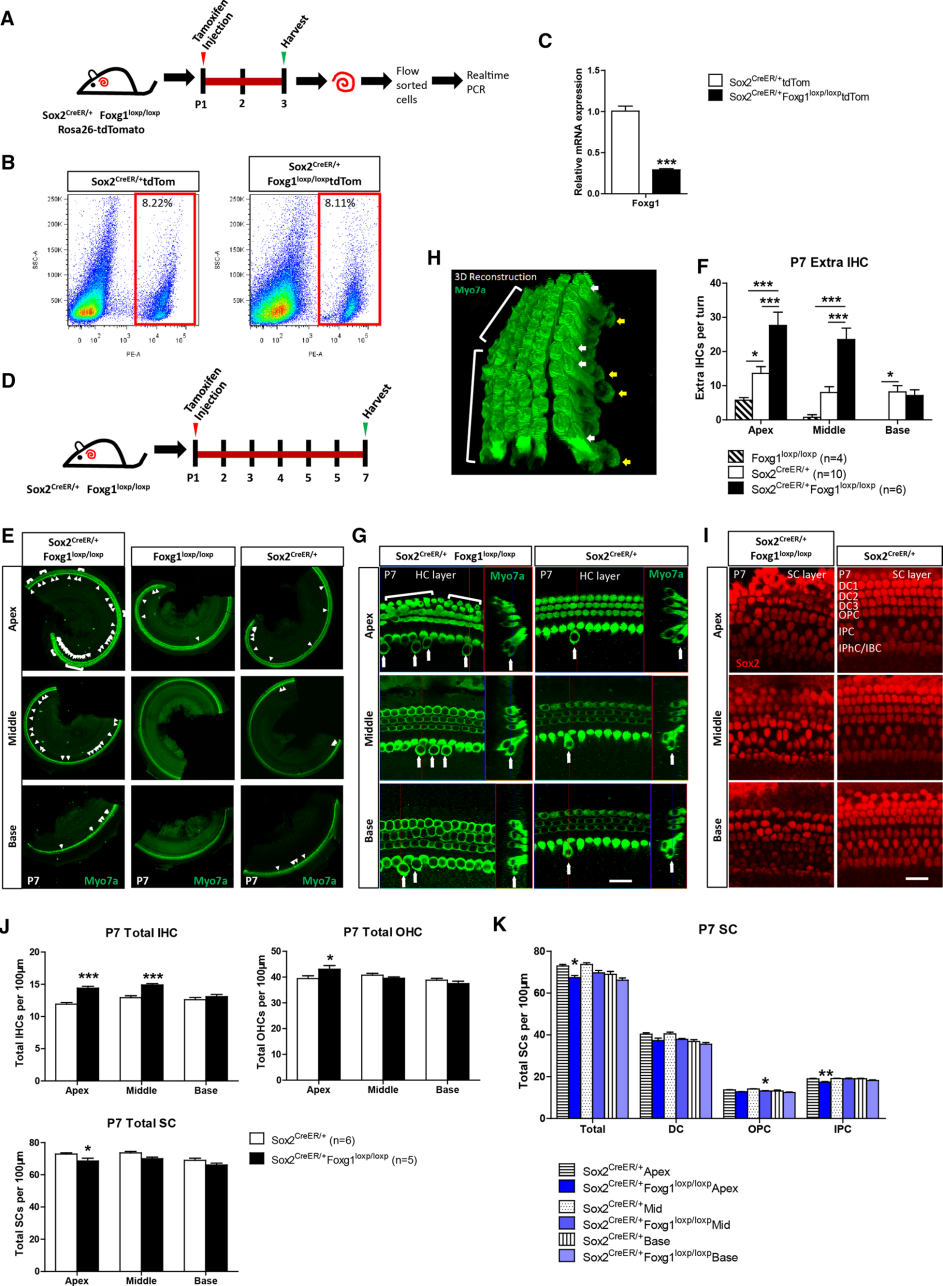
*Foxg1* cKD in Sox2+ SCs results in increased HC number and decreased SC number. **a** Tamoxifen was I.P. injected into P1 Sox2^CreER/+^ Foxg1^loxp/loxp^ Rosa26-tdTomato mice to knock down *Foxg1* in Sox2+ SCs, and the mice were sacrificed at P3 for FAC sorting of Sox2+ SCs for real-time qPCR. **b** FAC sorting data for Sox2+ SCs. **c** Quantification of *Foxg1* mRNA expression based on four independent qPCR experiments. ***p < 0.001. **d** Tamoxifen was I.P. injected into P1 Sox2^CreER/+^ Foxg1^loxp/loxp^ mice to knockdown *Foxg1* in Sox2+ SCs, and the mice were sacrificed at P7. **e**–**i** Extra IHCs (arrows) and OHCs (square brackets) are seen in the apical (Apex), middle (Middle), and basal (Base) turns of P7 Sox2^CreER/+^ Foxg1^loxp/loxp^ mice cochleae (**e**). Statistical analysis of the extra IHCs is shown in (**f**). The HC layer (**g**) and SC layer (**i**) are also shown. 3D reconstruction of extra HCs (white and yellow arrows) is shown in (**h**). Scale bar, 20 µm. Sox2^CreER/+^ mice and Foxg1^loxp/loxp^ mice were used as controls. Myo7a and Sox2 were used as HC and SC markers, respectively. (**j**, **k**) Quantification of the total IHCs, total OHCs, total SCs (**j**), and different kinds of SCs (**k**) per 100 µm cochlea length. The *n* refers to the number of mice. *p < 0.05, **p < 0.01, ***p < 0.001. *DC* Deiter’s cell. *OPC* outer pillar cell. *IPC* inner pillar cell. *IPhC* inner phalangeal cell. *IBC* inner border cell

For neonatal mice (P0–P7), the cochleae were dissected with sharp forceps (WPI) in cold HBSS and then fixed in 4% paraformaldehyde for 1 h at room temperature (RT). For mice older than P7, cochleae were fixed in 4% paraformaldehyde for 1 h, decalcified with 0.5 M EDTA for 1–3 days (depending on the age of the mice), both at RT, and then dissected in HBSS. The cochleae were washed with PBS and then blocked with blocking solution (5% donkey serum, 0.5% Triton X100, 0.02% sodium azide, and 1% bovine serum albumin in pH 7.4 PBS) for 1 h at RT. The cochleae were then incubated with primary antibodies diluted in PBT1 (2.5% donkey serum, 0.1% Triton X100, 0.02% sodium azide, and 1% bovine serum albumin in pH 7.4 PBS) at 4 °C overnight. After washing with 0.1% Triton X100 in pH 7.4 PBS for three times, the cochleae were incubated with fluorescence-conjugated secondary antibody (Invitrogen) or phalloidin (Invitrogen), both diluted 1:400 in PBT2 (0.1% Triton X100 and 1% bovine serum albumin in pH 7.4 PBS), for 1 h at RT. After another three times of washing, the cochleae were mounted in antifade fluorescence mounting medium (DAKO). The primary antibodies used were anti-Myosin7a (myo7a; Proteus Bioscience, #25-6790; DSHB, #138-1; both 1:1000 dilution in PBT1), anti-Sox2 (Santa Cruz Biotechnology, #17320, 1:400 dilution in PBT1), anti-Foxg1 (Abcam, #ab18259, 1:400 dilution in PBT1), anti-Ctbp2 (BD Biosciences, #612044, 1:400 dilution in PBT1), anti-PSD95 (Millipore, #MAB1596, 1:400 dilution in PBT1), and anti-Tuj1 (Neuromics, # MO15013, 1:400 dilution in PBT1). The Click-it EdU imaging kit (Invitrogen) was used after blocking to label proliferating cells. For FM1-43 staining, the cochleae were dissected, incubated with 4 µM FM1-43 (Thermo) at RT for 30 s, and then washed with PBS. TUNEL Kit (Roche) was used to detect apoptotic cells in cochleae of P7 *Foxg1* cKD mice and control mice according to the manufacturer’s instructions. For image acquisition, all images were scanned with a Zeiss microscope (LSM 710) with the same hardware settings, including laser intensity, gain, etc., to enable a direct comparison between treatment conditions. Because SCs are not always in the same layer, we performed Z projection with ImageJ software to catch all of the SCs for some of the SC layer images, including Figs. 1i and 4b. Also, because the nucleus of extra inner HCs (IHCs) were not in the same layer with the nucleus of regular IHCs as shown in the cross-section image in Figs. 1g, 3c, 6c and e, Fig. S5C and Fig. S7E, we performed Z projection with ImageJ software to catch the Ctbp2 and PSD95 staining images in Fig. 6c and Fig. S6A. The other images were all single confocal planes. To better show the location of the extra IHCs, we also performed 3D reconstruction with the Zeiss software in Figs. 1h and Fig. 6c.

### Data quantification

For most of the data quantification, such as total IHC number, total outer HC (OHC) number and total SC number, we randomly took one or two 20 × or 40 × low-magnification confocal images of the cochleae in each turn as representative images. The cochleae were always in the center of the image (320 µm or 160 µm cochlear length per image). We counted the number of total IHCs, OHCs or SCs in the image, averaged the results of two images for each turn and presented the data as per 100 µm. For SC quantification, we counted three rows of Deiter’s cells (DCs), inner pillar cells (IPCs) and outer pillar cells (OPCs). If the counting object was relatively rare and the randomly taken images would not represent the true counting result, such as EdU+ SC number and tdTomato+ HC number, we quantified the whole cochlea and present the data as per turn or per cochlea. For all experiments, the treatment conditions were blinded to the analyst. At least three mice were used for quantification, and only one ear of each mouse was analyzed.

### Sphere-forming assay

Lgr5-EGFP^CreER/+^ Foxg1^loxp/loxp^ mice and Lgr5-EGFP^CreER/+^ control mice were I.P. injected with tamoxifen at P1 and sacrificed at P3. The cochleae were dissected at P3 and then digested with trypsin into single cells for FAC sorting of Lgr5+ cells. The sorted Lgr5+ cells from *Foxg1* cKD mice and control mice were separately cultured at a density of 2 cells/μl in Costar ultra-low attachment dishes for 5 days in DMEM/F12 medium supplemented with N2 (1:100 dilution, Invitrogen), B27 (1:50 dilution, Invitrogen), heparin sulfate (50 ng/ml, Sigma), and the growth factors bFGF (10 ng/ml, Sigma), EGF (20 ng/ml, Sigma), and IGF-1 (50 ng/ml, Sigma). Spheres were then digested with trypsin into single cells and cultured in the same way for the next generation. Images of all the spheres in each well of each generation were taken with a Zeiss microscope (HAL 100) at the end of the culture, and the sphere numbers and diameters were quantified.

### Scanning electron microscopy (SEM)

As previously described [45], the cochleae were dissected, postfixed in 0.5% OsO_4_, dehydrated in ethanol, dried, and then coated with gold. A scanning electron microscope (FEI Quanta 200) operating at 15 kV was used to take images of the hair bundles.

### RNA extraction and real-time qPCR

Sox2^CreER/+^ Foxg1^loxp/loxp^ Rosa26-tdTomato mice and Sox2^CreER/+^ Rosa26-tdTomato mice were I.P. injected with tamoxifen at P1 and then sacrificed at P3. The cochleae were dissected and then digested with trypsin into single cells for FAC sorting of Sox2+ SCs. Approximately, 50,000 cochlear Sox2+ SCs from *Foxg1* cKD mice and control mice were used to extract total RNA with the GenElute™ Single Cell RNA Purification Kit (Sigma, #RNB300). RNA was reverse transcribed into cDNA, and real-time quantitative polymerase chain reaction (real-time qPCR) was performed using the FastStart Universal SYBR Green Master (ROX) kit (Roche) on a Bio-Rad C1000 Touch thermal cycler to quantify the gene expression levels. Real-time qPCR conditions were an initial denaturing step of 15 s at 95 °C followed by 40 cycles of 15 s denaturation at 95 °C, 60 s annealing at 60 °C, and 20 s extension at 72 °C. *Gapdh* was used as the reference endogenous gene, and gene expression was quantified using the ∆∆*C*_*T*_ method as follows: after obtaining the *C*_*T*_ value of the gene expression, we normalized these data to the *Gapdh* CT value (∆*C*_*T*_) to eliminate the sample differences (e.g., the small differences in cell number and so on). We next normalized the data to the control group data (∆∆*C*_*T*_) to compare the group differences, after which we calculated the $2^{{ - \Delta \Delta C_{T} }}$ value to quantify the fold difference between the control group and *Foxg1* cKD group. The real-time qPCR primers are shown in Supplementary Table 1.

### Statistical analysis

For each experimental condition, at least three independent experiments were performed, and the “*n*” in the figures refers to the number of mice, cell culture wells, or real-time qPCR experimental repetitions as illustrated in the figure legends. Data were analyzed using GraphPad Prism 6 software and are presented as means ± standard errors of the means. Two-tailed, unpaired Student’s *t* tests were used to determine statistical significance when comparing two groups, and two-way ANOVA followed by a Bonferroni post-test was used when comparing more than two groups. A value of *p* < 0.05 was considered statistically significant.

## Results

### *Foxg1* cKD in neonatal mouse cochlear SCs led to significantly greater numbers of HCs and fewer SCs

Foxg1 plays important roles in brain and eye development, especially in neuron differentiation, and *Foxg1* knock-out leads to inner ear malformation and multiple rows of HCs during embryonic development [44, 45], as well as the loss of clear OHC/IHC distinctions and reduced p75+ IPCs [29]. We speculated that Foxg1 might play an important role in HC regeneration; however, *Foxg1* knock-out mice die at birth, and thus the role of Foxg1 in HC regeneration after birth remains unclear. To investigate the role of Foxg1 in SCs, tamoxifen was I.P. injected into P1 Sox2^CreER/+^ Foxg1^loxp/loxp^ mice to induce the Cre enzyme activity and thus conditionally knockdown *Foxg1* in Sox2+ SCs (Fig. 1a and d). The Foxg1^loxp/loxp^ mice and Sox2^CreER/+^ mice were used as controls. Foxg1 was successfully down-regulated in the cochlear SCs of Sox2^CreER/+^ Foxg1^loxp/loxp^ mice (Fig. 1a–c and Fig. S2). P7 *Foxg1* cKD mice were sacrificed to find numerous extra IHCs in the apical, middle, and basal turns, and four rows of OHCs were also found in the apical turns (Fig. 1e, g and h). Although extra IHCs could also be seen in the cochleae of Sox2^CreER/+^ control mice due to Sox2 haploinsufficiency as reported recently [76, 77], statistical analysis showed that there were significantly more extra IHCs in the *Foxg1* cKD mice compared to the controls (Fig. 1f). We statistically analyzed the number of total IHCs, OHCs, and SCs per 100 μm cochlea length, and found significantly more IHCs in the cochleae of *Foxg1* cKD mice compared to Sox2^CreER/+^ control mice, and the number of extra IHCs decreased from the apical turns to the basal turns (Fig. 1f and j, Table S2). We also found four rows of OHCs in some parts of the apical turns of *Foxg1* cKD mice cochleae, and the statistical analysis showed a significant increase of apical OHC number (Fig. 1j, Table S2). As previously reported [29, 30], we also quantified the numbers of various cell types of SCs and found that the numbers of IPCs and OPCs were significantly decreased in the apical and middle turns of *Foxg1* cKD mice cochleae, respectively (Fig. 1i, j and k), which suggest that the extra HCs might be generated by direct trans-differentiation of SCs.

To further verify the role of Foxg1 in SCs, we also bred Sox9^CreER/+^ Foxg1^loxp/loxp^ mice to conditionally knockdown *Foxg1* in Sox9 + SCs. Similar to previous reports [78], we found that Sox9 is expressed in SCs of the cochlea (Fig. S3A). And Sox9^CreER/+^ Foxg1^loxp/loxp^ mice cochleae showed more extra HCs in the apical turns compared to Sox9^CreER/+^ control mice (Fig. S3A–C, Table S4), which is consistent with the phenotype of Sox2^CreER/+^ Foxg1^loxp/loxp^ mice. However, there were not significantly more extra HCs in the middle and basal turns of Sox9^CreER/+^ Foxg1^loxp/loxp^ mice cochleae compared to Sox9^CreER/+^ control mice, which might be due to the relative low Cre efficiency in Sox9-CreER mice.

To determine the initial Cre induction ratio and the initial tdTomato labeling of Sox2^CreER/+^ Rosa26-tdTomato mice, Sox9^CreER/+^ Rosa26-tdTomato mice, and Lgr5-EGFP^CreER/+^ Rosa26-tdTomato mice, we measured how many HCs were labeled by tdTomato when tamoxifen was injected at P1 or P3 and the mice were sacrificed 48 h later at P3 or P5, respectively, in which tdTomato only labeled the original Sox2+, Lgr5+ and Sox9+ cells at P1 or P3 but not the subsequently generated HCs. For Sox2^CreER/+^ Rosa26-tdTomato mice, at P3 only some of the HCs in the apex tip and part of the apex were labeled by tdTomato (Fig. S7A, the yellow bracket in ① and the white bracket in ②), and in the rest of the apical turns and all of the middle and basal turns. Only very few HCs were labeled by tdTomato (as indicated by white arrowheads). Fig. S7B shows the higher magnification of the apex tip, apex, middle, and base of the P3 cochlea. At P5, only a small number of HCs in the apex tip (the white bracketed region) was labeled by tdTomato, and we found very few tdTomato+ HCs (white arrows) in the apical, middle, and basal turns (Fig. S7C and D). Thus, only a small part of the apical HCs in Sox2^CreER/+^ Rosa26-tdTomato mice cochleae was originally labeled by tdTomato when tamoxifen was injected at P1 and P3. More importantly, in all the experiments we used Sox2^CreER/+^ Rosa26-tdTomato mice as the controls. Sox2^CreER/+^ Foxg1^loxp/loxp^ Rosa26-tdTomato mice were compared with Sox2^CreER/+^ Rosa26-tdTomato controls, and the originally labeled tdTomato+ HCs also appeared in the controls, thus the increased number of tdTomato+ HCs was only because of *Foxg1* cKD in SCs. For Lgr5-EGFP^CreER/+^ and Sox9^CreER/+^ mice, we also injected tamoxifen at P1 and sacrificed the mice at P3. Lgr5-EGFP^CreER/+^ Rosa26-tdTomato results are shown in Fig. S7E, and Sox9^CreER/+^ Rosa26-tdTomato results are shown in Fig.S3A. We did not find any tdTomato+ HCs in either of these mice, and the tdTomato+ cells were restricted to Lgr5+ progenitors and Sox9+ SCs.

### The extra IHCs survived at least to P30

**Fig. 2:**
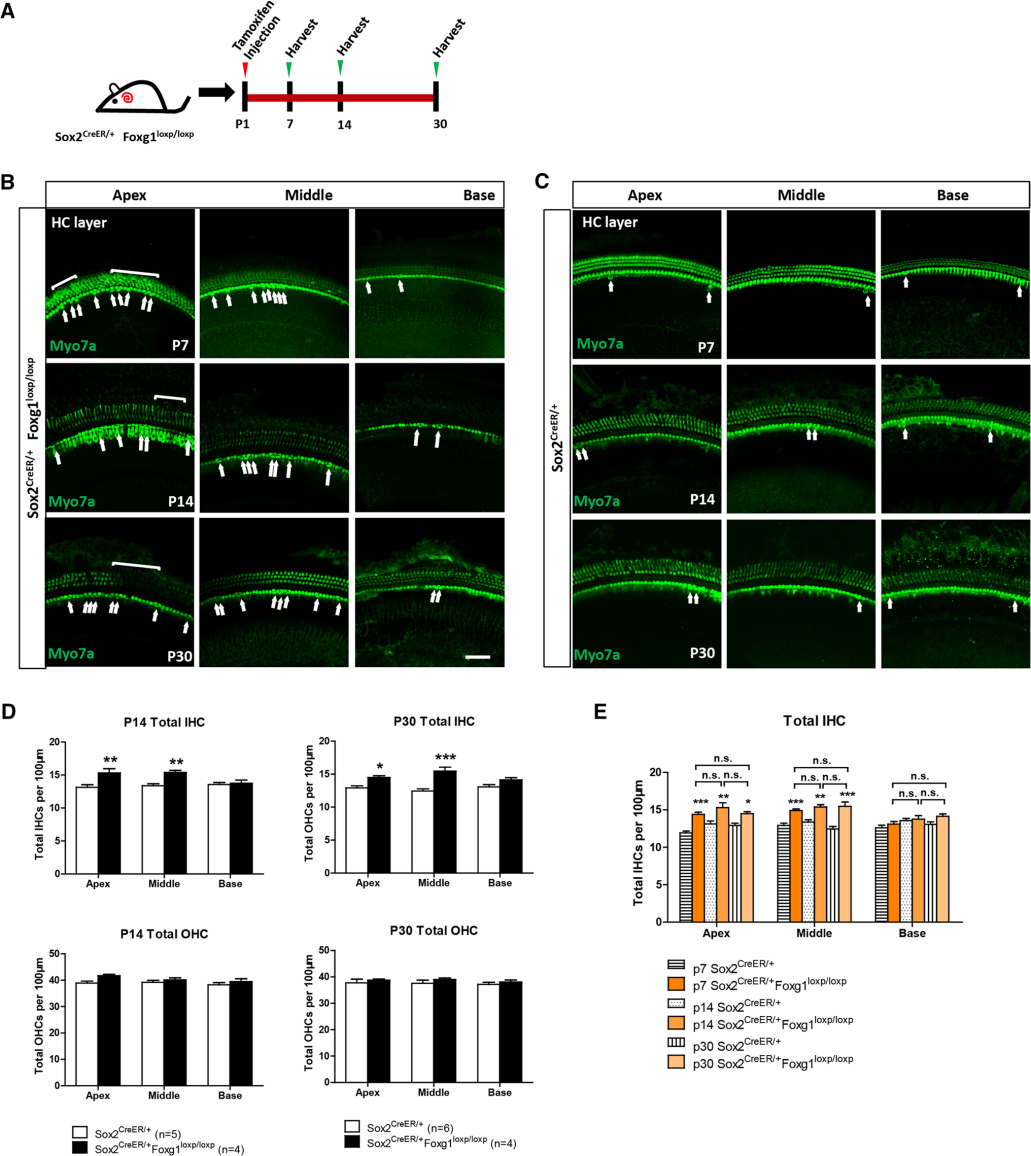
The extra IHCs could survive to P30. **a** Tamoxifen was I.P. injected at P1, and the mice were sacrificed at P7, P14, and P30. **b**, **c** Extra IHCs (arrows) and OHCs (square brackets) are seen in the apical (Apex), middle (Middle), and basal (Base) turns of P7 Sox2^CreER/+^ Foxg1^loxp/loxp^ mice cochleae. Sox2^CreER/+^ mice were used as controls. Myo7a was used as the HC marker. Scale bar, 50 µm. (**d**, **e**) Quantification of the total IHCs and OHCs per 100 µm cochlea length at P14 and P30 (**d**) and the comparison between the three ages in control and *Foxg1* cKD mice (**e**). The *n* refers to the number of mice. *p < 0.05, **p < 0.01, ***p < 0.001*, n.s*. not significant

It was previously reported that some of the newly regenerated HCs will progressively die [10]. Thus, we also analyzed the survival of the extra IHCs in *Foxg1* cKD mice cochleae and found that in P7, P14, and P30 mice the extra IHCs still existed in the cKD cochleae (Fig. 2a–c). The statistical analysis showed that the number of extra IHCs was not significantly changed from P7 to P30 (Fig. 2d and e), which suggests that the extra IHCs could survive at least to P30. We also observed that P30 *Foxg1* cKD mice were significantly smaller than the control mice (Fig. S4A and B). The ABR test showed that the low-frequency (4 kHz and 8 kHz) hearing thresholds of the *Foxg1* cKD mice were significantly increased (Fig. S4C), which might due to the extra IHCs in the apical turns.

To verify whether the extra HCs can be generated at a later age, we injected mice with tamoxifen at P7 and sacrificed them at P14 and analyzed the HCs. We found very few extra HCs at P14 in both Sox2^CreER/+^ Foxg1^loxp/loxp^ mice and Sox2^CreER/+^ control mice, and there was no significant difference in HC number between the two groups (Fig. S5A and B). This is consistent with previous reports that SCs rapidly lose the ability to regenerate HCs after P7 [2–4, 9], and suggests that the majority of the extra HCs existing at P30 are mainly generated before P7.

### *Foxg1* cKD in neonatal mouse cochlear Lgr5+ progenitors led to significantly more IHCs that could survive at least to P30

**Fig. 3:**
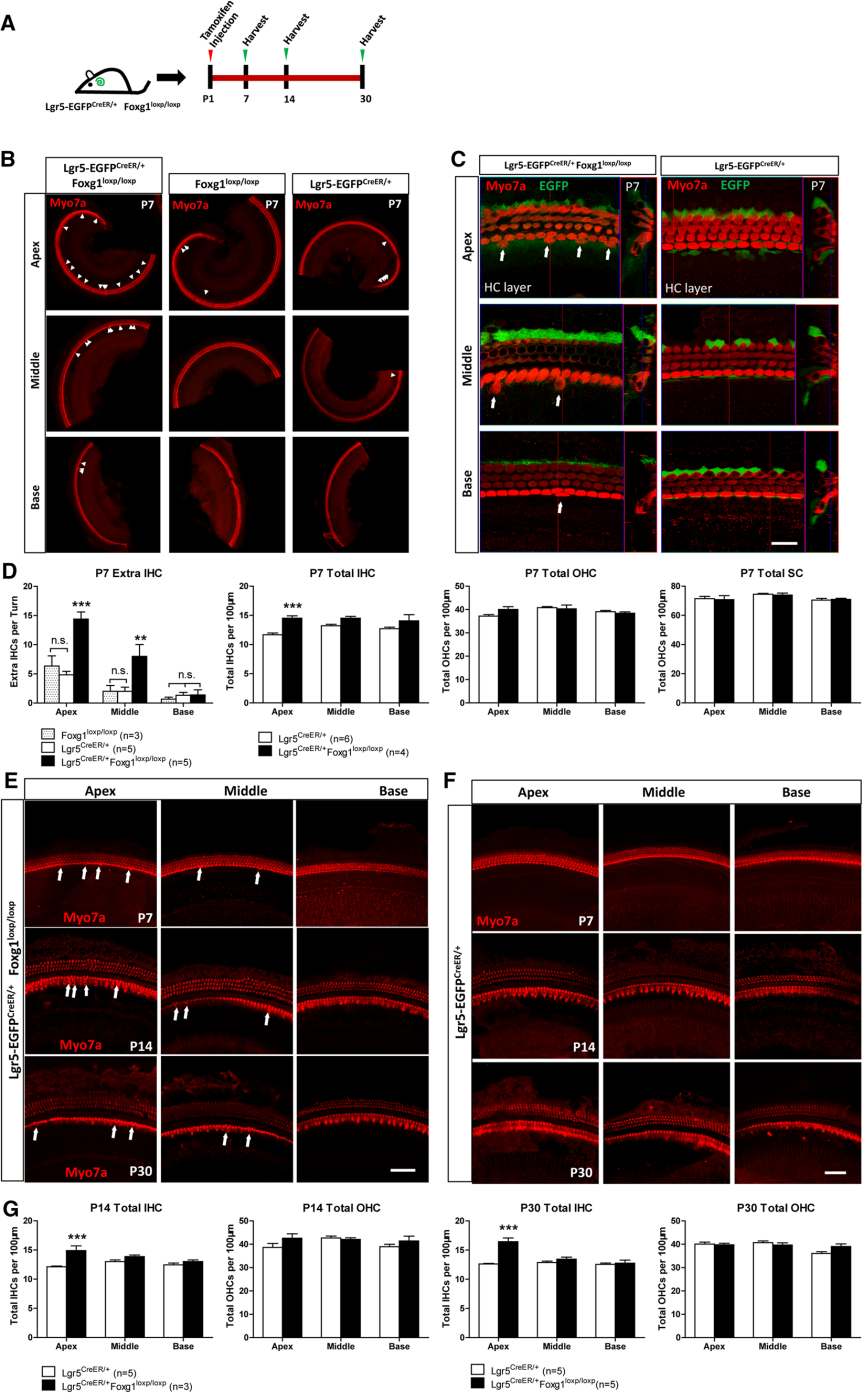
*Foxg1* cKD in Lgr5+ progenitors results in an increased number of IHCs that could survive to P30. **a** Tamoxifen was I.P. injected into P1 Lgr5-EGFP^CreER/+^ Foxg1^loxp/loxp^ mice to knockdown *Foxg1* in Lgr5+ progenitors, and the mice were sacrificed at P7, P14, and P30. **b**, **c** Extra IHCs (arrows) are seen in the apical (Apex), middle (Middle), and basal (Base) turns of P7 Lgr5-EGFP^CreER/+^ Foxg1^loxp/loxp^ mice cochleae. Lgr5-EGFP^CreER/+^ mice and Foxg1^loxp/loxp^ mice were used as controls. Myo7a was used as the HC marker. Scale bar, 20 µm. (**d**) Quantification of the extra IHCs, total IHCs, total OHCs, and total SCs. *n* is the number of mice. *p < 0.05, **p < 0.01, ***p < 0.001. **e**, **f** Extra IHCs (arrows) are seen in the apical (Apex), middle (Middle), and basal (Base) turns of P7, P14, and P30 Lgr5-EGFP^CreER/+^ Foxg1^loxp/loxp^ mice cochleae. Lgr5-EGFP^CreER/+^ mice were used as controls. Myo7a was used as the HC marker. Scale bar, 50 µm. **g** Quantification of the total IHCs and OHCs per 100 µm cochlea length at P14 and P30 in control and *Foxg1* cKD mice. The *n* refers to the number of mice. *p < 0.05, **p < 0.01, ***p < 0.001

To determine whether *Foxg1* cKD in Lgr5+ progenitors also leads to extra HCs, we crossed Lgr5-EGFP-CreERT2 mice with Foxg1-floxp mice to generate Lgr5-EGFP^CreER/+^ Foxg1^loxp/loxp^ double-positive mice. To activate the Cre enzyme, tamoxifen was I.P. injected into P1 mice, and the cochleae were dissected at P7, P14, and P30 (Fig. 3a). Lgr5-EGFP^CreER/+^ mice and Foxg1^loxp/loxp^ mice were used as the controls. A significant number of extra IHCs were also found in P7 Lgr5-EGFP^CreER/+^ Foxg1^loxp/loxp^ double-positive mice compared to the control mice and the number of extra IHCs decreased from apical turns to basal turns (Fig. 3b–d). We also quantified the number of total IHCs, OHCs, and SCs per 100 μm cochlea length at P7 and found significantly more IHCs in the cochleae of *Foxg1* cKD mice compared to the Lgr5^CreER/+^ control mice (Fig. 3d). However, the statistical analysis showed no significant increase of OHC number and no significant decrease of SC number (Fig. 3d, Table S3). Significant more extra IHCs were still found in both P14 and P30 *Foxg1* cKD mice compared to the control mice (Fig. 3e–g). These results suggest that *Foxg1* cKD in Lgr5+ progenitors also induces the generation of extra IHCs in neonatal mouse cochlea.

### *Foxg1* cKD in neonatal mouse cochlear SCs and progenitors did not significantly change their proliferation ability in vivo and in vitro

**Fig. 4:**
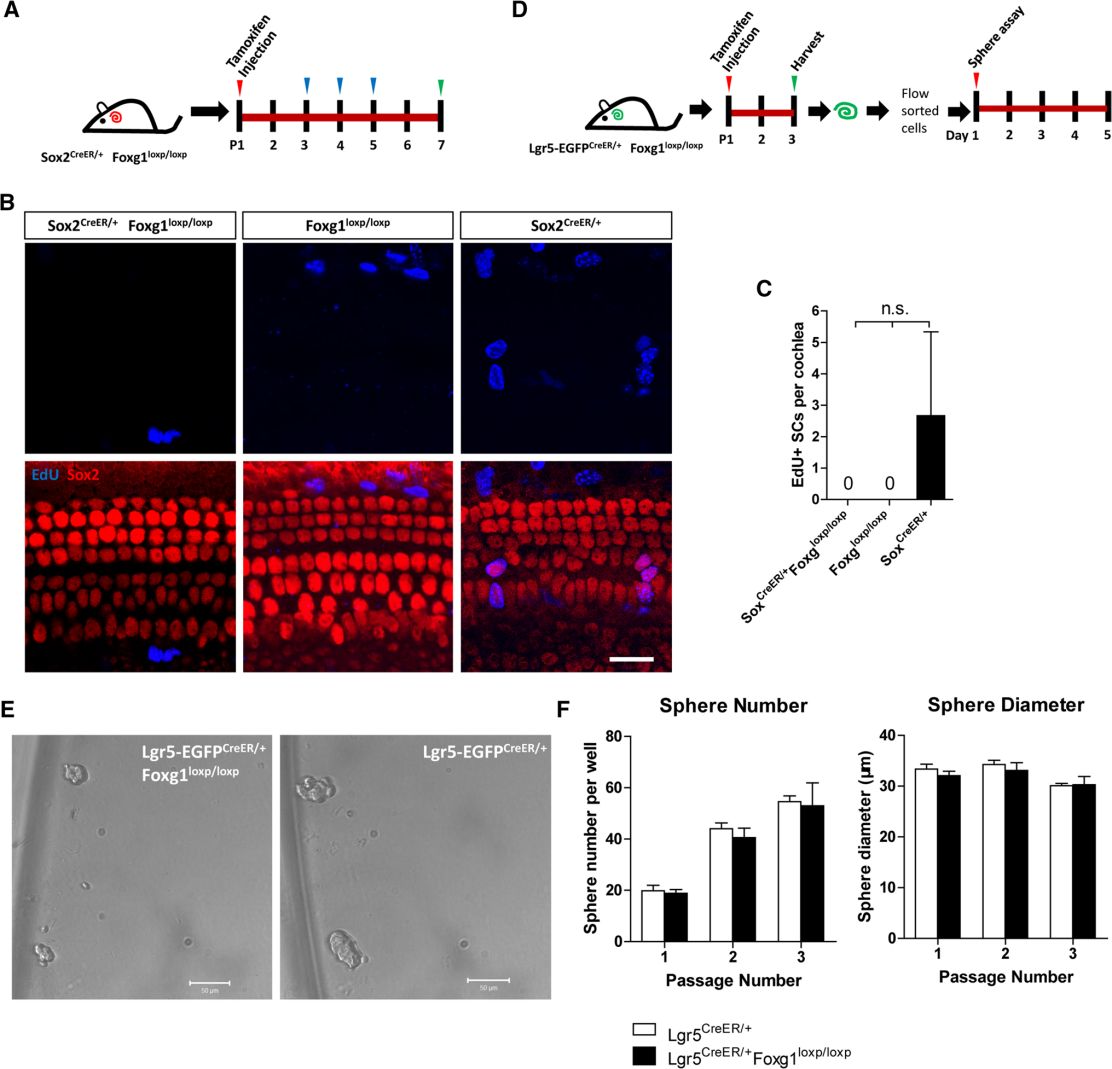
The proliferation of Sox2+ SCs and Lgr5+ progenitors has no change in *Foxg1* cKD mice. **a** EdU (50 mg/kg body weight) was injected at P3, P4, and P5 to label proliferating cells. **b** EdU was stained (blue) in Sox2^CreER/+^ Foxg1^loxp/loxp^, Foxg1^loxp/loxp^, and Sox2^CreER/+^ mice. Myo7a and Sox2 were used as HC and SC markers, respectively. Scale bar, 20 µm. **c** Quantification of EdU+ SCs per cochlea. *n* = 3 mice per group. *n.s*. not significant. **d** Tamoxifen was injected into Lgr5-EGFP^CreER/+^ Foxg1^loxp/loxp^ mice to conditionally knockdown *Foxg1* in Lgr5+ progenitors. After 2 days, Lgr5+ progenitors were isolated by FAC sorting and cultured in vitro for 5 days to form spheres. **e** Spheres formed by Lgr5+ progenitors from Lgr5-EGFP^CreER/+^ Foxg1^loxp/loxp^ and Lgr5-EGFP^CreER/+^ mice. Scale bar, 50 µm. **f** Quantification of sphere number per well and sphere diameter of each passage. At least three wells of spheres were quantified. *n.s*. not significant

The generation of extra HCs might be the result of mitotic HC generation, direct trans-differentiation of SCs to HCs, or both. To determine the mechanism behind the generation of extra HCs in *Foxg1* cKD mice, we I.P. injected tamoxifen to Sox2^CreER/+^ Foxg1^loxp/loxp^ double-positive mice at P1, and then I.P. injected EdU (50 mg/kg body weight) from P3 to P5 to mark proliferating cells (Fig. 4a). Mice were sacrificed at P7, and EdU was detected using the Click-it EdU imaging kit. However, we failed to detect any EdU+/Sox2+ SCs in any of the three Sox2^CreER/+^ Foxg1^loxp/loxp^ mice cocleae (Fig. 4b), indicating that the new HCs might not be generated by mitotic generation. We used both Sox2^CreER/+^ mice and Foxg1^loxp/loxp^ mice as the controls and treated it the same way. We did not find any EdU+ SCs in any of the three Foxg1^loxp/loxp^ mice or in two of the three Sox2^CreER/+^ mice. We only found a few EdU+ SCs in the third Sox2^CreER/+^ mouse. However, the statistical analysis showed no significant difference (Fig. 4b and c).

To verify whether *Foxg1* cKD will lead to apoptosis in SCs, we performed a TUNEL assay to measure apoptosis of SCs in *Foxg1* cKD mice cochleae. At P7, we did not detect any TUNEL + cells among the IPCs, OPCs, or three rows of DCs in either *Foxg1* cKD mice or Sox2^CreER/+^ control mice, while only a few TUNEL + cells were found among the Hensen cells in both mice (Fig. S5C). The quantification results showed no significant difference in the number of TUNEL+ Hensen cells between *Foxg1* cKD mice (8.5 ± 2.5 per 100 μm) and Sox2^CreER/+^ control mice (9.5 ± 1.5 per 100 μm). This suggests that the decrease of cochlear SC number in *Foxg1* cKD mice probably is not caused by apoptosis of SCs.

To further evaluate the effect of Foxg1 in regulating the proliferation and sphere-forming ability of Lgr5+ progenitors, Lgr5-EGFP ^CreER/+^ Foxg1^loxp/loxp^ mice and Lgr5-EGFP ^CreER/+^ control mice were I.P. injected with tamoxifen at P1 and sacrificed at P3. The cochleae were dissected and then digested with trypsin for FAC sorting of Lgr5+ progenitors. The sorted Lgr5+ progenitors were then cultured in vitro to form spheres which were then passaged for three generations (Fig. 4d). The sphere-forming assays showed that Lgr5+ progenitors of *Foxg1* cKD mice showed no significant differences in either sphere number or sphere diameter of all three generations compared to the control mice (Fig. 4d–f), suggesting that *Foxg1* cKD in Lgr5+ progenitors does not significantly affect the proliferation and sphere-forming ability of Lgr5+ progenitors.

### The extra HCs in *Foxg1* cKD mouse cochlea originated from Sox2+ SCs in the neonatal mouse cochlea

**Fig. 5:**
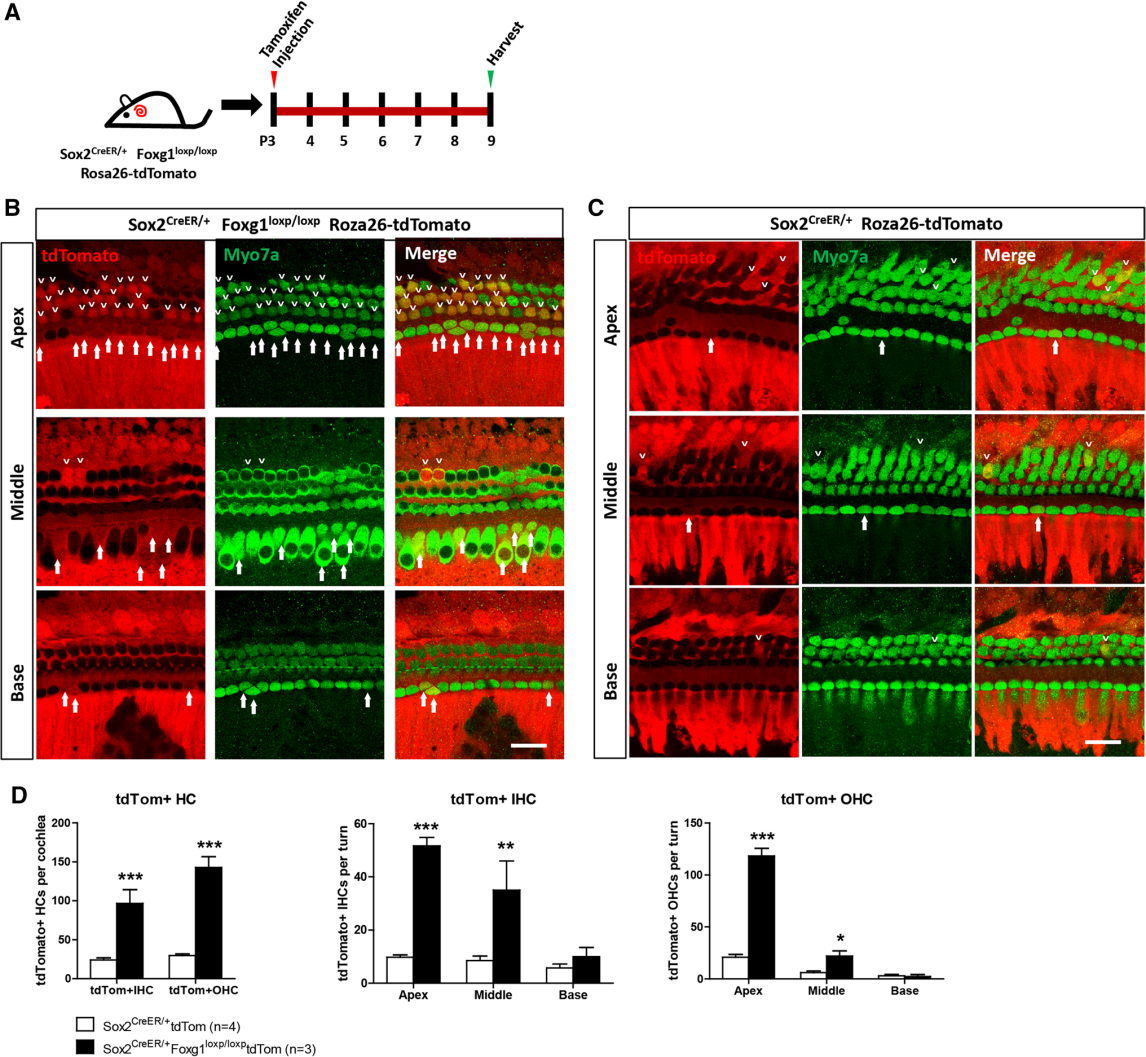
Lineage tracing of Sox2+ SCs. **a** Tamoxifen was injected at P3, and Sox2+ SCs were traced by following the expression of tdTomato fluorescent protein. **b**, **c** Lineage tracing images of cochlear Sox2+ SCs in Sox2^CreER/+^ Foxg1^loxp/loxp^ Rosa26-tdTomato mice (**b**) and Sox2^CreER/+^ Rosa26-tdTomato mice (**c**). tdTomato+/Myo7a+ IHCs and OHCs are indicated by arrows and arrowheads, respectively. Scale bar, 20 µm. **d** Quantification of tdTomato+ (Tom+) IHCs and OHCs per cochlea and per turn. The *n* refers to the number of mice. *p < 0.05

Next, we crossed Sox2^CreER/+^ Foxg1^loxp/loxp^ double-positive mice with Foxg1^loxp/loxp^ Rosa26-tdTomato mice to generate Sox2^CreER/+^ Foxg1^loxp/loxp^ Rosa26-tdTomato triple-positive *Foxg1* cKD mice to lineage trace Sox2+ SCs (Fig. 5a). Sox2 ^CreER/+^ Rosa26-tdTomato mice were used as the controls. We found significantly more tdTomato+ IHCs and OHCs in the apical and middle turns of triple-positive mice cochleae than that of control mice cochleae (Fig. 5b–d). These results suggest that the extra HCs in *Foxg1* cKD mice cochleae originate from Sox2+ SCs and *Foxg1* cKD increase the HC regeneration and SC differentiation. Considering all the results we showed above, *Foxg1* cKD promotes HC regeneration and leads to large numbers of extra HCs probably by mainly inducing direct trans-differentiation rather than mitotic HC generation.

### The extra IHCs had normal hair bundles, synapse number, FM1-43+ mechano-transduction (MET) channels, and innervation

**Fig. 6:**
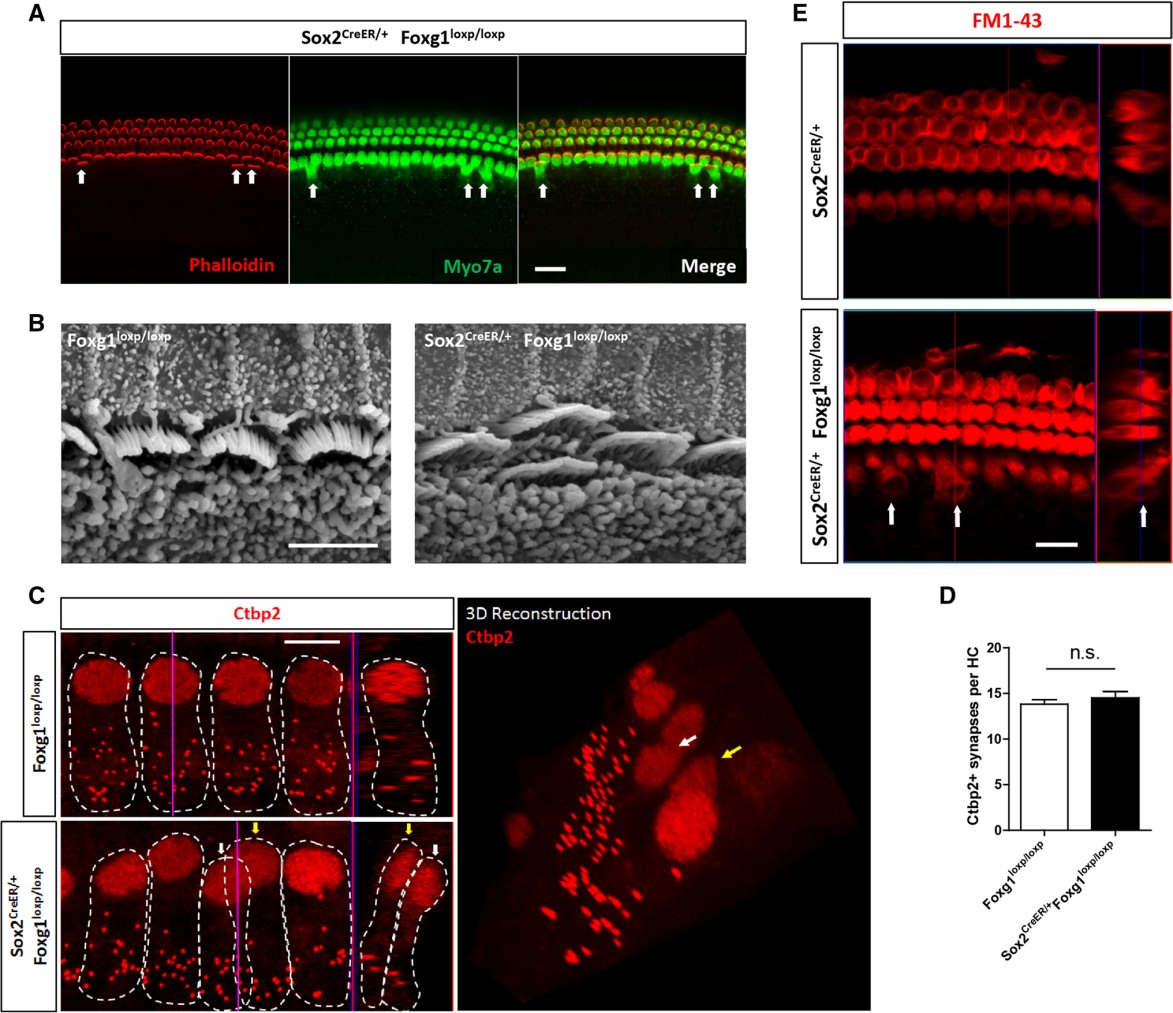
Hair bundle, synapse, and FM1-43 staining of the extra IHCs. **a** Phalloidin was used to stain the hair bundles of the HCs. Extra IHCs are indicated by arrows. Scale bar, 20 µm. **b** Hair bundles of the extra IHCs by SEM. Scale bar, 5 µm. **c** Ctbp2 was used to stain synapses (dotted staining) of IHCs. Each IHC and its Ctbp2+ synapses are indicated by dotted white circles. Extra IHCs (white arrows) and normal IHCs (yellow arrows) are shown in both confocal images and 3D reconstructions. **d** Quantification of the synapse number of IHCs. *n* = 5 mice per group. *n.s.* not significant. **e** FM1-43 dye was up taken by extra IHCs (white arrows). Scale bar, 5 µm

To confirm whether the newly formed extra IHCs in *Foxg1* cKD mice cochleae have normal HC characteristics, we investigated the hair bundles and the synapse number of the extra IHCs. We used phalloidin to stain the hair bundles and found that the extra IHCs had normal hair bundles (Fig. 6a). SEM also showed normal hair bundles of the extra IHCs (Fig. 6b). Next, we used Ctbp2 to stain the synapses of the IHCs and found that the extra IHCs also had normal synapse number, similar to the control IHCs (Fig. 6c and d). We also used FM1-43 to verify whether the extra IHCs have functional MET channels, and found that the extra IHCs were all FM1-43+, just like the normal IHCs (Fig. 6e), suggesting that the extra IHCs also have the ability to uptake FM1-43 dye and have functional MET channels. Moreover, to directly show the innervation of the extra IHCs with spiral ganglion neurons, we used Ctbp2 and PSD95 to label the pre- and post-synapse, respectively, and found that normal IHCs and extra IHCs had similar numbers of innervated synapses (Fig. S6A and B). We also used Tuj1 to label the axons that innervate the IHCs and found that all the extra IHCs had neuronal axons branching to them (Fig. S6C). Together, these results suggest that the extra IHCs have normal IHC functions as we investigated.

### Characterization of gene expression changes in *Foxg1* cKD mice cochlear SCs by real-time qPCR

**Fig. 7:**
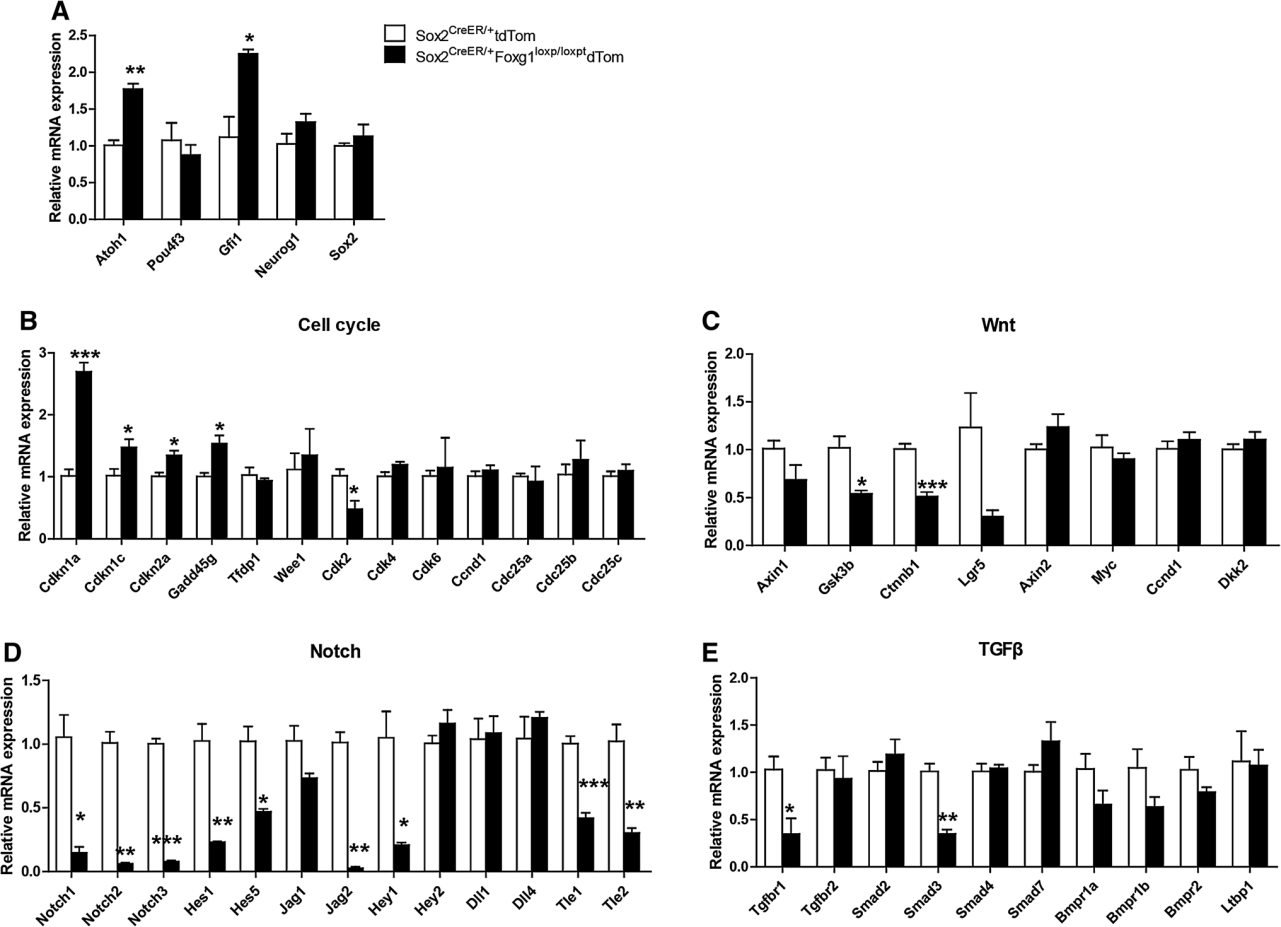
Expression quantification of related genes and signaling pathways in *Foxg1* cKD mice cochlear SCs. **a–e** Relative mRNA expression patterns of genes related to HC differentiation (**a**), cell cycle (**b**), and Wnt signaling (**c**), Notch signaling (**d**), and TGFβ signaling pathways (**e**). Four independent qPCR experiments were performed. *p < 0.05, **p < 0.01, ***p < 0.001

To determine the mechanism through which Foxg1 is involved in HC regeneration, we used Sox2^CreER/+^ Foxg1^loxp/loxp^ Rosa26-tdTomato triple-positive mice and Sox2^CreER/+^ Rosa26-tdTomato control mice to isolate the tdTomato+/Sox2+ SCs by flow cytometry, and the mRNA was then extracted for real-time qPCR to quantify the related gene expression level (Fig. 1a and b). As expected, *Foxg1* was down-regulated in *Foxg1* cKD SC**s** (Fig. 1c). The mRNA expression of *Atoh1* and *Gfi1*, two transcription factors that regulate HC generation, was up-regulated (Fig. 7a), which is consistent with our experimental results. However, the mRNA expression of the other important factors *Pou4f3*, *Neurog1*, and *Sox2* did not change.

Considering that Foxg1 has been reported to regulate genes involved in the TGFβ, Notch, and Wnt signaling pathways as well as some cell cycle genes [35–43, 79], we analyzed these pathways by real-time qPCR. To determine the effect of *Foxg1* cKD on proliferation-related signaling pathways, we quantified the mRNA expression levels of some important cell cycling genes and Wnt signaling genes. We found that the cell cycle repressors *Cdkn1a*, *Cdkn1c*, *Cdkn2a*, and *Gadd45* *g* were all up-regulated and cell cycle-dependent kinase *Cdk2* was down-regulated in *Foxg1* cKD SCs (Fig. 7b). The expression of most Wnt signaling pathway genes did not change significantly, while only β-catenin (*Ctnnb1*) and *Gsk3β* were down-regulated (Fig. 7c). Our results presented above showed that *Foxg1* cKD might lead to extra HCs by promoting the direct trans-differentiation of SCs. Thus, we checked two cell differentiation-related pathways, the Notch and TGFβ signaling pathways. We found that many genes of the Notch signaling pathway, such as *Notch 1*-*3*, *Hes1*, *Hes5*, *Jag2*, and *Hey1*, were significantly down-regulated (Fig. 7d). The Notch-related transcription factors, *Tle1* and *Tle2*, were also down-regulated (Fig. 7d). However, the expression of most TGFβ signaling pathway genes did not change significantly, while only *Tgfbr1* and *Smad3* were down-regulated (Fig. 7e). All these results suggest that *Foxg1* cKD in SCs probably leads to the generation of new HCs mainly through down-regulation of the cell cycle pathway and the Notch signaling pathway.

## Discussion

It is known that a limited number of HCs can be regenerated in newborn mice from SCs and inner ear progenitor cells, and several studies have shown that many important signaling pathways are involved in HC regeneration, such as Wnt, Notch, and Shh [7, 8, 11–14, 80–84]. Many other genes and related pathways have also been shown to play important roles in HC regeneration [85, 86], and these pathways might have crosstalk with each other to affect the proliferation and differentiation of SCs and Lgr5+ progenitors [13, 14]. Foxg1, one of the FOX protein family members, plays important roles in brain, eye, and ear development [24, 35, 36, 44, 45, 55, 57, 60, 64, 66]. In the inner ear, previous studies showed that during embryonic development *Foxg1* knock-out mice have shortened cochleae with multiple extra rows of HCs [44, 45]. However, *Foxg1*-null mice show hypoplasia of the telencephalon, abnormal eye and ear development, and die soon after birth [44, 45, 55], thus the role of Foxg1 in HC regeneration in the postnatal mouse cochlea is still unclear. In this study, we found that *Foxg1* cKD in both Sox2+ SCs and Lgr5+ progenitors led to significant numbers of extra HCs, especially extra IHCs that could survive at least to P30. The extra IHCs had normal hair bundles and synapses. Moreover, *Foxg1* cKD failed to induce the proliferation of SCs, and lineage tracing data showed that more tdTomato+ HCs originated from Sox2+ SCs in the cKD mouse cochlea, and thus the new extra HCs were most likely generated by direct trans-differentiation of SCs. Real-time qPCR data showed that cell cycle genes and the Notch signaling pathway might be involved in this process.

The role of Foxg1 has been characterized mainly in forebrain development [58–61], and the absence of Foxg1 leads to structural defects of both the dorsal and ventral telencephalon due to reduced proliferation and premature differentiation of neuroepithelial cells [55]. In cortical progenitor cells, Foxg1 promotes self-renewal of neural precursors and inhibits neuronal differentiation [55, 59, 62, 63]. The dynamic expression of *Foxg1* during cortical development is essential for the proper assembly of the cerebral cortex, and *Foxg1* is down-regulated when progenitors undergo neuronal differentiation and up-regulated when differentiating neurons integrate into the cortical plate [64]. In postnatal mice, Foxg1 also plays important roles in maintaining the hippocampal dentate gyrus progenitor pool, and the lack of Foxg1 promotes both gliogenesis and neurogenesis [24]. The results of these studies are consistent with our findings that *Foxg1* cKD increased the differentiation of SCs and led to the generation of extra HCs. However, we did not observe any significant differences in sphere number or sphere diameter in *Foxg1* cKD Lgr5+ progenitors, suggesting that Foxg1 might have no significant effects in regulating the proliferation of Lgr5+ progenitors in the postnatal mouse cochlea. In one of the three Sox2^CreER/+^ mice, we could find a few EdU+ SCs, while we could not find any EdU+ SCs in any of the three Sox2^CreER/+^ Foxg1^loxp/loxp^ mice, three Foxg1^loxp/loxp^ mice or the other two Sox2^CreER/+^ mice. Though the statistical analysis showed no significant difference, we suspect that *Foxg1* cKD might slightly decrease the proliferation of neonatal mouse cochlear SCs.

Pauley et al. reported the embryonic phenotype of the *Foxg1*-null mouse cochlea in which they showed that *Foxg1*-null mice have shortened cochleae and multiple rows of extra HCs and SCs [45]. Our results showing that *Foxg1* cKD in both SCs and Lgr5+ progenitors results in significantly more HCs in neonatal mice cochleae which are consistent with their results in embryonic mice. They also suspected that Notch signaling might be involved in this process, and this hypothesis is supported by our results. However, they found multiple rows of SCs, while we found that *Foxg1* cKD in SCs led to decreased numbers of SCs. This might be because Foxg1 plays different roles during different development stages.

One recent report showed that *Sox2* haploinsufficiency (Sox2-CreER, Sox2-EGFP, in which one allele of the *Sox2* gene is replaced by CreER or EGFP such that Sox2 is expressed at only half of the normal expression level) also increases the IHC number in vivo [76, 77]. Thus in our study, we also used Sox2^CreER/+^ mice as the control to avoid overestimating the effect of cKD of *Foxg1*. The statistical analysis showed that although Sox2^CreER/+^ mice also had some extra IHCs, Sox2^CreER/+^ Foxg1^loxp/loxp^ mice had significantly more extra HCs than Sox2^CreER/+^ mice (Fig. 1e–g). Moreover, there were significantly more newly generated HCs (Myo7a+/tdTomato+ cells) in Sox2^CreER/+^ Foxg1^loxp/loxp^ Rosa26-tdTomato mice than that in Sox2^CreER/+^ Rosa26-tdTomato mice (Fig. 5). To verify this finding, we used two other CreER lines—Lgr5-EGFP^CreER/+^ mice and Sox9^CreER/+^ mice. In one experiment, we used Lgr5-EGFP^CreER/+^ mice as the control and found that Lgr5-EGFP^CreER/+^ Foxg1^loxp/loxp^ mice had many more extra IHCs in the apical and middle turns compared with Lgr5-EGFP^CreER/+^ mice (Fig. 3b–d). In the other experiment, we used Sox9^CreER/+^ Foxg1^loxp/loxp^ mice to further verify the effects of Foxg1 in SCs, and we found that these mice also had many more extra HCs in the apical turns compared with Sox9^CreER/+^ control mice (Fig. S3B and C). These results all suggest that cKD of *Foxg1* in SCs leads to the extra HCs. However, when we quantified the SC number in Lgr5-EGFP^CreER/+^ Foxg1^loxp/loxp^ mice and Sox9^CreER/+^ Foxg1^loxp/loxp^ mice, we found that the average number of apical SCs was smaller in Lgr5-EGFP^CreER/+^ Foxg1^loxp/loxp^ mice than that in Lgr5-EGFP^CreER/+^ control mice (70.78 ± 2.76 and 71.46 ± 1.55 per 100 μm, respectively, Table S3), and the average number of apical SCs was smaller in Sox9^CreER/+^ Foxg1^loxp/loxp^ mice than that in Sox9^CreER/+^ control mice (71.13 ± 1.02 and 71.67 ± 2.66 per 100 μm, respectively, Table S4), but these differences were not statistically significant. This might because the Cre efficiency of Lgr5-EGFP^CreER/+^ and Sox9^CreER/+^ is not as high as Sox2^CreER/+^, which was demonstrated by the greater number of extra HCs in Sox2^CreER/+^ Foxg1^loxp/loxp^ mice than that in Lgr5-EGFP^CreER/+^ Foxg1^loxp/loxp^ mice and Sox9^CreER/+^ Foxg1^loxp/loxp^ mice. Thus, the decreased SC numbers of Lgr5-EGFP^CreER/+^ Foxg1^loxp/loxp^ mice and Sox9^CreER/+^ Foxg1^loxp/loxp^ mice were also much lower than that of Sox2^CreER/+^ Foxg1^loxp/loxp^ mice, and the decreased SC number of Lgr5-EGFP^CreER/+^ Foxg1^loxp/loxp^ mice and Sox9^CreER/+^ Foxg1^loxp/loxp^ mice was too few to result in the total SC number significantly decreased in Lgr5-EGFP^CreER/+^ Foxg1^loxp/loxp^ mice and Sox9^CreER/+^ Foxg1^loxp/loxp^ mice.

During embryonic development, Foxg1 plays important roles in neurogenesis through crosstalk with many other signaling pathways that also regulate neuronal progenitor proliferation and neuronal differentiation [38, 39, 42, 43, 87]. Foxg1 represses TGFβ-induced neuronal differentiation and associates with the FoxO/Smad complex to regulate cell cycle progression in early developmental stages [40, 42, 43]. Foxg1 is also involved in the regulation of progenitor cell differentiation in the telencephalon by interacting with the Notch signaling pathway factors Hes1 and Groucho/TLE [39]. Foxg1 coordinates the activity of the Shh pathway and Wnt/β-catenin pathway by acting as a downstream effector of the Shh pathway and as a direct transcriptional repressor of Wnt ligands [37]. Foxg1 was also reported to suppress the Wnt/β-catenin pathway to restrict tissue development [35, 36] and to directly repress the cell cycle repressor *Cdkn1a* [40, 42, 79]. In addition, altered cellular interactions change the detailed mosaic pattern of the organ of Corti, which was recently demonstrated in a model of *Atoh1* replacement with *Neurog1* [31].

Because the TGFβ, Notch, and Wnt pathways and some cell cycle repressors were reported to have a crosstalk with Foxg1, we analyzed these pathways by real-time qPCR. We found that the most obvious expression changes were among genes in the Notch pathway and genes of cell cycle repressors, while the expression of most genes in the TGFβ and Wnt pathways was not significantly altered by cKD of *Foxg1*. Many previous studies have suggested that Notch is a very important pathway involved in HC regeneration [5, 8, 12–14, 19, 24, 74, 84, 88], and down-regulation of the Notch signaling pathway in the *Foxg1* cKD SCs might be one of the important mechanisms leading to the phenotype of extra HCs. *Hes1*, *Hes5*, and *Hey1* are three of the important Notch downstream transcription factors, and knock-out of *Hes1*, *Hes5*, and *Hey1* in the inner ear also results in extra HCs [89–92], which is consistent with our results, and thus down-regulation of *Hes1*, *Hes5*, and *Hey1* by *Foxg1* cKD might contribute to the phenotype of extra HCs. Also, Hes and Hey were reported to regulate HC differentiation by regulating the *Atoh1* promoter [93, 94], which is also consistent with *Atoh1* up-regulation in *Foxg1* cKD mice cochleae (Fig. 7a). One recent work demonstrated that replacement of one allele of *Atoh1* by *Neurog1* combined with a self-terminating second *Atoh1* allele rescued most IHCs and some OHCs as compared with the massive loss of IHCs in the Atoh1-Cre; Atoh1^f/f^ mouse [31]. However, we did not find any expression changes of *Neurog1* in *Foxg1* cKD SCs (Fig. 7a), which suggests that the phenotype of *Foxg1* cKD in SCs might not involve Neurog1. The lateral inhibition of Notch receptors plays important roles in inner ear development and HC regeneration [21, 23–25], and three of the Notch receptors, *Notch1*–*3*, were down-regulated in *Foxg1* cKD SCs. TLEs are involved in the gene regulatory functions of a variety of signaling pathways, including Notch and Wnt signaling [22, 95]. Groucho/TLE1 inhibits neuron differentiation [96], and Foxg1 is involved in the regulation of progenitor cell differentiation in the telencephalon by interacting with Groucho/TLE and Hes [39, 79, 97]. *Tle1* and *Tle2* were both down-regulated by *Foxg1* cKD (Fig. 7d), which suggests that TLEs might play important roles in HC regeneration. *Jag2*, one of the Notch ligands, was also down-regulated by *Foxg1* cKD, and null mutation of the *Jag2* gene was reported to cause supernumerary HC differentiation in the cochleae [98, 99], which is consistent with our results. The cell cycle repressor *Cdkn1a*, which is a downstream target of *Foxg1*, was up-regulated in *Foxg1* cKD SCs. The other cell cycle repressors *Cdkn1c*, *Cdkn2a*, and *Gadd45* *g* were also up-regulated, while cell cycle-dependent kinase *Cdk2* was down-regulated. These results suggest that cell cycle pathway is repressed to some extent in *Foxg1* cKD SCs. However, we did not observe any significant decrease of the proliferative ability of *Foxg1* cKD SCs or Lgr5+ progenitors (Fig. 4), which might be due to the overall combined effects of other genes.

In summary, we specifically knocked down *Foxg1* in Sox2+ SCs and Lgr5+ progenitors of neonatal mice cochleae and found that this resulted in significantly more HCs. Because we found reduced numbers of SCs and no obviously proliferating SCs, and because we lineage traced more tdTomato+ HCs after cKD of *Foxg1*, we hypothesize that *Foxg1* cKD probably leads to the generation of extra HCs through direct trans-differentiation of SCs and progenitors into HCs. In addition, the real-time qPCR results showed that some cell cycle repressors were up-regulated, while genes involved in the Notch signaling pathway were significantly down-regulated in *Foxg1* cKD SCs, which might contribute to the generation of extra HCs in *Foxg1* cKD mice cochleae.

## Electronic supplementary material

Below is the link to the electronic supplementary material.

Supplementary material 1 (DOCX 34 kb)

Supplementary material 2 (TIFF 386 kb)

Supplementary material 3 (TIFF 4192 kb)

Supplementary material 4 (TIFF 2720 kb)

Supplementary material 5 (TIFF 1806 kb)

Supplementary material 6 (TIFF 2362 kb)

Supplementary material 7 (TIFF 5324 kb)

Supplementary material 8 (TIFF 7519 kb)

## References

[1] Rubel EW, Furrer SA, Stone JS (2013). A brief history of hair cell regeneration research and speculations on the future. Hear Res.

[2] Bramhall NF, Shi F, Arnold K, Hochedlinger K, Edge AS (2014). Lgr5-positive supporting cells generate new hair cells in the postnatal cochlea. Stem Cell Rep.

[3] Shi F, Kempfle JS, Edge AS (2012). Wnt-responsive Lgr5-expressing stem cells are hair cell progenitors in the cochlea. J Neurosci.

[4] Chai R, Kuo B, Wang T, Liaw EJ, Xia A, Jan TA, Liu Z, Taketo MM, Oghalai JS, Nusse R, Zuo J, Cheng AG (2012). Wnt signaling induces proliferation of sensory precursors in the postnatal mouse cochlea. Proc Natl Acad Sci USA.

[5] Li W, You D, Chen Y, Chai R, Li H (2016). Regeneration of hair cells in the mammalian vestibular system. Front Med.

[6] Uchimura T, Hollander JM, Nakamura DS, Liu Z, Rosen CJ, Georgakoudi I, Zeng L (2017). An essential role for IGF2 in cartilage development and glucose metabolism during postnatal long bone growth. Development.

[7] Zhang Y, Guo L, Lu X, Cheng C, Sun S, Li W, Zhao L, Lai C, Zhang S, Yu C, Tang M, Chen Y, Chai R, Li H (2018). Characterization of Lgr6+ cells as an Enriched population of hair cell progenitors compared to Lgr5+ cells for hair cell generation in the neonatal mouse cochlea. Front Mol Neurosci.

[8] You D, Guo L, Li W, Sun S, Chen Y, Chai R, Li H (2018). Characterization of Wnt and Notch-responsive Lgr5+ hair cell progenitors in the striolar region of the neonatal mouse utricle. Front Mol Neurosci.

[9] Bermingham-McDonogh O, Reh TA (2011). Regulated reprogramming in the regeneration of sensory receptor cells. Neuron.

[10] Cox BC, Chai R, Lenoir A, Liu Z, Zhang L, Nguyen DH, Chalasani K, Steigelman KA, Fang J, Rubel EW, Cheng AG, Zuo J (2014). Spontaneous hair cell regeneration in the neonatal mouse cochlea in vivo. Development.

[11] Lu X, Sun S, Qi J, Li W, Liu L, Zhang Y, Chen Y, Zhang S, Wang L, Miao D, Chai R, Li H (2017). Bmi1 regulates the proliferation of cochlear supporting cells via the canonical Wnt signaling pathway. Mol Neurobiol.

[12] Waqas M, Zhang S, He Z, Tang M, Chai R (2016). Role of Wnt and Notch signaling in regulating hair cell regeneration in the cochlea. Front Med.

[13] Ni W, Zeng S, Li W, Chen Y, Zhang S, Tang M, Sun S, Chai R, Li H (2016). Wnt activation followed by Notch inhibition promotes mitotic hair cell regeneration in the postnatal mouse cochlea. Oncotarget.

[14] Wu J, Li W, Lin C, Chen Y, Cheng C, Sun S, Tang M, Chai R, Li H (2016). Co-regulation of the Notch and Wnt signaling pathways promotes supporting cell proliferation and hair cell regeneration in mouse utricles. Sci Rep.

[15] Pryazhnikov E, Mugantseva E, Casarotto P, Kolikova J, Fred SM, Toptunov D, Afzalov R, Hotulainen P, Voikar V, Terry-Lorenzo R, Engel S, Kirov S, Castren E, Khiroug L (2018). Longitudinal two-photon imaging in somatosensory cortex of behaving mice reveals dendritic spine formation enhancement by subchronic administration of low-dose ketamine. Sci Rep.

[16] Hu L, Lu J, Chiang H, Wu H, Edge AS, Shi F (2016). Diphtheria toxin-induced cell death triggers Wnt-dependent hair cell regeneration in neonatal mice. J Neurosci.

[17] Abate G, Colazingari S, Accoto A, Conversi D, Bevilacqua A (2018). Dendritic spine density and EphrinB2 levels of hippocampal and anterior cingulate cortex neurons increase sequentially during formation of recent and remote fear memory in the mouse. Behav Brain Res.

[18] Ni W, Zeng S, Li W, Chen Y, Zhang S, Tang M, Sun S, Chai R, Li H (2016). Wnt activation followed by Notch inhibition promotes mitotic hair cell regeneration in the postnatal mouse cochlea. Oncotarget.

[19] Li W, Wu J, Yang J, Sun S, Chai R, Chen ZY, Li H (2015). Notch inhibition induces mitotically generated hair cells in mammalian cochleae via activating the Wnt pathway. Proc Natl Acad Sci USA.

[20] Liu Q, Gibson MP, Sun H, Qin C (2013). Dentin sialophosphoprotein (DSPP) plays an essential role in the postnatal development and maintenance of mouse mandibular condylar cartilage. J Histochem Cytochem.

[21] Zhu G, Ye R, Jung DY, Barron E, Friedline RH, Benoit VM, Hinton DR, Kim JK, Lee AS (2013). GRP78 plays an essential role in adipogenesis and postnatal growth in mice. FASEB J.

[22] Galabova-Kovacs G, Catalanotti F, Matzen D, Reyes GX, Zezula J, Herbst R, Silva A, Walter I, Baccarini M (2008). Essential role of B-Raf in oligodendrocyte maturation and myelination during postnatal central nervous system development. J Cell Biol.

[23] Cunningham D, DeBarber AE, Bir N, Binkley L, Merkens LS, Steiner RD, Herman GE (2015). Analysis of hedgehog signaling in cerebellar granule cell precursors in a conditional Nsdhl allele demonstrates an essential role for cholesterol in postnatal CNS development. Hum Mol Genet.

[24] Tian C, Gong Y, Yang Y, Shen W, Wang K, Liu J, Xu B, Zhao J, Zhao C (2012). Foxg1 has an essential role in postnatal development of the dentate gyrus. J Neurosci.

[25] Xia F, Dohi T, Martin NM, Raskett CM, Liu Q, Altieri DC (2011). Essential role of the small GTPase Ran in postnatal pancreatic islet development. PLoS One.

[26] Jahan I, Pan N, Fritzsch B (2015). Opportunities and limits of the one gene approach: the ability of Atoh1 to differentiate and maintain hair cells depends on the molecular context. Front Cell Neurosci.

[27] Chonko KT, Jahan I, Stone J, Wright MC, Fujiyama T, Hoshino M, Fritzsch B, Maricich SM (2013). Atoh1 directs hair cell differentiation and survival in the late embryonic mouse inner ear. Dev Biol.

[28] Nichols DH, Pauley S, Jahan I, Beisel KW, Millen KJ, Fritzsch B (2008). Lmx1a is required for segregation of sensory epithelia and normal ear histogenesis and morphogenesis. Cell Tissue Res.

[29] Jahan I, Elliott KL, Fritzsch B (2018). Understanding molecular evolution and development of the organ of corti can provide clues for hearing restoration. Integr Comp Biol.

[30] Booth KT, Azaiez H, Jahan I, Smith RJH, Fritzsch B (2018). Intracellular regulome variability along the organ of corti: evidence, approaches, challenges, and perspective. Front Genet.

[31] Jahan I, Pan N, Kersigo J, Fritzsch B (2015). Neurog1 can partially substitute for Atoh1 function in hair cell differentiation and maintenance during organ of Corti development. Development.

[32] Pan N, Jahan I, Kersigo J, Kopecky B, Santi P, Johnson S, Schmitz H, Fritzsch B (2011). Conditional deletion of Atoh1 using Pax2-Cre results in viable mice without differentiated cochlear hair cells that have lost most of the organ of Corti. Hear Res.

[33] Jahan I, Pan N, Kersigo J, Fritzsch B (2010). Neurod1 suppresses hair cell differentiation in ear ganglia and regulates hair cell subtype development in the cochlea. PLoS One.

[34] Jahan I, Kersigo J, Pan N, Fritzsch B (2010). Neurod1 regulates survival and formation of connections in mouse ear and brain. Cell Tissue Res.

[35] Smith R, Huang YT, Tian T, Vojtasova D, Mesalles-Naranjo O, Pollard SM, Pratt T, Price DJ, Fotaki V (2017). The transcription factor Foxg1 promotes optic fissure closure in the mouse by suppressing Wnt8b in the nasal optic stalk. J Neurosci.

[36] Fotaki V, Smith R, Pratt T, Price DJ (2013). Foxg1 is required to limit the formation of ciliary margin tissue and Wnt/beta-catenin signalling in the developing nasal retina of the mouse. Dev Biol.

[37] Danesin C, Peres JN, Johansson M, Snowden V, Cording A, Papalopulu N, Houart C (2009). Integration of telencephalic Wnt and hedgehog signaling center activities by Foxg1. Dev Cell.

[38] Adesina AM, Veo BL, Courteau G, Mehta V, Wu X, Pang K, Liu Z, Li XN, Peters L (2015). FOXG1 expression shows correlation with neuronal differentiation in cerebellar development, aggressive phenotype in medulloblastomas, and survival in a xenograft model of medulloblastoma. Hum Pathol.

[39] Yao J, Lai E, Stifani S (2001). The winged-helix protein brain factor 1 interacts with groucho and hes proteins to repress transcription. Mol Cell Biol.

[40] Vezzali R, Weise SC, Hellbach N, Machado V, Heidrich S, Vogel T (2016). The FOXG1/FOXO/SMAD network balances proliferation and differentiation of cortical progenitors and activates Kcnh3 expression in mature neurons. Oncotarget.

[41] Siegenthaler JA, Miller MW (2008). Generation of Cajal-Retzius neurons in mouse forebrain is regulated by transforming growth factor beta-Fox signaling pathways. Dev Biol.

[42] Seoane J, Le HV, Shen L, Anderson SA, Massague J (2004). Integration of Smad and forkhead pathways in the control of neuroepithelial and glioblastoma cell proliferation. Cell.

[43] Dou C, Lee J, Liu B, Liu F, Massague J, Xuan S, Lai E (2000). BF-1 interferes with transforming growth factor beta signaling by associating with Smad partners. Mol Cell Biol.

[44] Hwang CH, Simeone A, Lai E, Wu DK (2009). Foxg1 is required for proper separation and formation of sensory cristae during inner ear development. Dev Dyn.

[45] Pauley S, Lai E, Fritzsch B (2006). Foxg1 is required for morphogenesis and histogenesis of the mammalian inner ear. Dev Dyn.

[46] Macova I, Pysanenko K, Chumak T, Dvorakova M, Bohuslavova R, Syka J, Fritzsch B, Pavlinkova G (2019). Neurod1 is essential for the primary tonotopic organization and related auditory information processing in the midbrain. J Neurosci.

[47] Solomon KS, Logsdon JM, Fritz A (2003). Expression and phylogenetic analyses of three zebrafish FoxI class genes. Dev Dyn.

[48] Benayoun BA, Caburet S, Veitia RA (2011). Forkhead transcription factors: key players in health and disease. Trends Genet.

[49] Jackson BC, Carpenter C, Nebert DW, Vasiliou V (2010). Update of human and mouse forkhead box (FOX) gene families. Hum Genomics.

[50] Katoh M, Katoh M (2004). Human FOX gene family (Review). Int J Oncol.

[51] Katoh M, Igarashi M, Fukuda H, Nakagama H, Katoh M (2013). Cancer genetics and genomics of human FOX family genes. Cancer Lett.

[52] Ariani F, Hayek G, Rondinella D, Artuso R, Mencarelli MA, Spanhol-Rosseto A, Pollazzon M, Buoni S, Spiga O, Ricciardi S, Meloni I, Longo I, Mari F, Broccoli V, Zappella M, Renieri A (2008). FOXG1 is responsible for the congenital variant of Rett syndrome. Am J Hum Genet.

[53] Florian C, Bahi-Buisson N, Bienvenu T (2012). FOXG1-related disorders: from clinical description to molecular genetics. Mol Syndromol.

[54] Hebert JM, McConnell SK (2000). Targeting of cre to the Foxg1 (BF-1) locus mediates loxP recombination in the telencephalon and other developing head structures. Dev Biol.

[55] Xuan S, Baptista CA, Balas G, Tao W, Soares VC, Lai E (1995). Winged helix transcription factor BF-1 is essential for the development of the cerebral hemispheres. Neuron.

[56] Kersigo J, D’Angelo A, Gray BD, Soukup GA, Fritzsch B (2011). The role of sensory organs and the forebrain for the development of the craniofacial shape as revealed by Foxg1-cre-mediated microRNA loss. Genesis.

[57] Huh S, Hatini V, Marcus RC, Li SC, Lai E (1999). Dorsal-ventral patterning defects in the eye of BF-1-deficient mice associated with a restricted loss of shh expression. Dev Biol.

[58] Hanashima C, Li SC, Shen L, Lai E, Fishell G (2004). Foxg1 suppresses early cortical cell fate. Science.

[59] Hanashima C, Shen L, Li SC, Lai E (2002). Brain factor-1 controls the proliferation and differentiation of neocortical progenitor cells through independent mechanisms. J Neurosci.

[60] Martynoga B, Morrison H, Price DJ, Mason JO (2005). Foxg1 is required for specification of ventral telencephalon and region-specific regulation of dorsal telencephalic precursor proliferation and apoptosis. Dev Biol.

[61] Vyas A, Saha B, Lai E, Tole S (2003). Paleocortex is specified in mice in which dorsal telencephalic patterning is severely disrupted. J Comp Neurol.

[62] Ahlgren S, Vogt P, Bronner-Fraser M (2003). Excess FoxG1 causes overgrowth of the neural tube. J Neurobiol.

[63] Brancaccio M, Pivetta C, Granzotto M, Filippis C, Mallamaci A (2010). Emx2 and Foxg1 inhibit gliogenesis and promote neuronogenesis. Stem Cells.

[64] Miyoshi G, Fishell G (2012). Dynamic FoxG1 expression coordinates the integration of multipolar pyramidal neuron precursors into the cortical plate. Neuron.

[65] Herrera E, Marcus R, Li S, Williams SE, Erskine L, Lai E, Mason C (2004). Foxd1 is required for proper formation of the optic chiasm. Development.

[66] Pratt T, Tian NM, Simpson TI, Mason JO, Price DJ (2004). The winged helix transcription factor Foxg1 facilitates retinal ganglion cell axon crossing of the ventral midline in the mouse. Development.

[67] Kawauchi S, Santos R, Kim J, Hollenbeck PL, Murray RC, Calof AL (2009). The role of foxg1 in the development of neural stem cells of the olfactory epithelium. Ann N Y Acad Sci.

[68] Kawauchi S, Kim J, Santos R, Wu HH, Lander AD, Calof AL (2009). Foxg1 promotes olfactory neurogenesis by antagonizing Gdf11. Development.

[69] Duggan CD, DeMaria S, Baudhuin A, Stafford D, Ngai J (2008). Foxg1 is required for development of the vertebrate olfactory system. J Neurosci.

[70] Chai R, Xia A, Wang T, Jan TA, Hayashi T, Bermingham-McDonogh O, Cheng AG (2011). Dynamic expression of Lgr5, a Wnt target gene, in the developing and mature mouse cochlea. J Assoc Res Otolaryngol.

[71] Lai B, Li M, Hu W, Li W, Gan WB (2018). The Phosphodiesterase 9 inhibitor PF-04449613 promotes dendritic spine formation and performance improvement after motor learning. Dev Neurobiol.

[72] Shen Y, Schlessinger K, Zhu X, Meffre E, Quimby F, Levy DE, Darnell JE (2004). Essential role of STAT3 in postnatal survival and growth revealed by mice lacking STAT3 serine 727 phosphorylation. Mol Cell Biol.

[73] Cai Y, Sun Z, Jia H, Luo H, Ye X, Wu Q, Xiong Y, Zhang W, Wan J (2017). Rpph1 upregulates CDC42 expression and promotes hippocampal neuron dendritic spine formation by competing with miR-330-5p. Front Mol Neurosci.

[74] Li WY, Wu JF, Yang JM, Sun S, Chai RJ, Chen ZY, Li HW (2015). Notch inhibition induces mitotically generated hair cells in mammalian cochleae via activating the Wnt pathway. Proc Natl Acad Sci USA.

[75] Chen Y, Li L, Ni W, Zhang Y, Sun S, Miao D, Chai R, Li H (2015). Bmi1 regulates auditory hair cell survival by maintaining redox balance. Cell Death Dis.

[76] Atkinson PJ, Dong Y, Gu S, Liu W, Najarro EH, Udagawa T, Cheng AG (2018). Sox2 haploinsufficiency primes regeneration and Wnt responsiveness in the mouse cochlea. J Clin Invest.

[77] Dabdoub A, Puligilla C, Jones JM, Fritzsch B, Cheah KS, Pevny LH, Kelley MW (2008). Sox2 signaling in prosensory domain specification and subsequent hair cell differentiation in the developing cochlea. Proc Natl Acad Sci USA.

[78] Mak AC, Szeto IY, Fritzsch B, Cheah KS (2009). Differential and overlapping expression pattern of SOX2 and SOX9 in inner ear development. Gene Expr Patterns.

[79] Verginelli F, Perin A, Dali R, Fung KH, Lo R, Longatti P, Guiot MC, Del Maestro RF, Rossi S, di Porzio U, Stechishin O, Weiss S, Stifani S (2013). Transcription factors FOXG1 and Groucho/TLE promote glioblastoma growth. Nat Commun.

[80] Cheng C, Guo L, Lu L, Xu X, Zhang S, Gao J, Waqas M, Zhu C, Chen Y, Zhang X, Xuan C, Gao X, Tang M, Chen F, Shi H, Li H, Chai R (2017). Characterization of the transcriptomes of Lgr5+ hair cell progenitors and Lgr5- supporting cells in the mouse cochlea. Front Mol Neurosci.

[81] Zhang S, Zhang Y, Yu P, Hu Y, Zhou H, Guo L, Xu X, Zhu X, Waqas M, Qi J, Zhang X, Liu Y, Chen F, Tang M, Qian X, Shi H, Gao X, Chai R (2017). Characterization of Lgr5+ progenitor cell transcriptomes after neomycin injury in the neonatal mouse cochlea. Front Mol Neurosci.

[82] Chen Y, Lu X, Guo L, Ni W, Zhang Y, Zhao L, Wu L, Sun S, Zhang S, Tang M, Li W, Chai R, Li H (2017). Hedgehog signaling promotes the proliferation and subsequent hair cell formation of progenitor cells in the neonatal mouse cochlea. Front Mol Neurosci.

[83] Waqas M, Guo L, Zhang S, Chen Y, Zhang X, Wang L, Tang M, Shi H, Bird PI, Li H, Chai R (2016). Characterization of Lgr5+ progenitor cell transcriptomes in the apical and basal turns of the mouse cochlea. Oncotarget.

[84] Ni W, Lin C, Guo L, Wu J, Chen Y, Chai R, Li W, Li H (2016). Extensive supporting cell proliferation and mitotic hair cell generation by in vivo genetic reprogramming in the neonatal mouse cochlea. J Neurosci.

[85] Zhang S, Zhang Y, Yu P, Hu Y, Zhou H, Guo L, Xu X, Zhu X, Waqas M, Qi J, Zhang X, Liu Y, Chen F, Tang M, Qian X, Shi H, Gao X, Chai R (2017). Characterization of Lgr5+ progenitor cell transcriptomes after neomycin injury in the neonatal mouse cochlea. Front Mol Neurosci.

[86] Waqas M, Guo L, Zhang S, Chen Y, Zhang X, Wang L, Tang M, Shi H, Bird PI, Li H, Chai R (2016). Characterization of Lgr5+ progenitor cell transcriptomes in the apical and basal turns of the mouse cochlea. Oncotarget.

[87] Arden KC (2004). FoxO: linking new signaling pathways. Mol Cell.

[88] Mizutari K, Fujioka M, Hosoya M, Bramhall N, Okano HJ, Okano H, Edge AS (2013). Notch inhibition induces cochlear hair cell regeneration and recovery of hearing after acoustic trauma. Neuron.

[89] Zheng JL, Shou J, Guillemot F, Kageyama R, Gao WQ (2000). Hes1 is a negative regulator of inner ear hair cell differentiation. Development.

[90] Zine A, Aubert A, Qiu J, Therianos S, Guillemot F, Kageyama R, de Ribaupierre F (2001). Hes1 and Hes5 activities are required for the normal development of the hair cells in the mammalian inner ear. J Neurosci.

[91] Li S, Mark S, Radde-Gallwitz K, Schlisner R, Chin MT, Chen P (2008). Hey2 functions in parallel with Hes1 and Hes5 for mammalian auditory sensory organ development. BMC Dev Biol.

[92] Tateya T, Imayoshi I, Tateya I, Ito J, Kageyama R (2011). Cooperative functions of Hes/Hey genes in auditory hair cell and supporting cell development. Dev Biol.

[93] Abdolazimi Y, Stojanova Z, Segil N (2016). Selection of cell fate in the organ of Corti involves the integration of Hes/Hey signaling at the Atoh1 promoter. Development.

[94] Su YX, Hou CC, Yang WX (2015). Control of hair cell development by molecular pathways involving Atoh1, Hes1 and Hes5. Gene.

[95] Robson LG, Di Foggia V, Radunovic A, Bird K, Zhang X, Marino S (2011). Bmi1 is expressed in postnatal myogenic satellite cells, controls their maintenance and plays an essential role in repeated muscle regeneration. PLoS One.

[96] Pan D, Rubin GM (1997). Kuzbanian controls proteolytic processing of Notch and mediates lateral inhibition during Drosophila and vertebrate neurogenesis. Cell.

[97] Hao J, Koesters R, Bouchard M, Gridley T, Pfannenstiel S, Plinkert PK, Zhang L, Praetorius M (2012). Jagged1-mediated Notch signaling regulates mammalian inner ear development independent of lateral inhibition. Acta Otolaryngol.

[98] Holmen SL, Zylstra CR, Mukherjee A, Sigler RE, Faugere MC, Bouxsein ML, Deng L, Clemens TL, Williams BO (2005). Essential role of beta-catenin in postnatal bone acquisition. J Biol Chem.

[99] Tschopp O, Yang ZZ, Brodbeck D, Dummler BA, Hemmings-Mieszczak M, Watanabe T, Michaelis T, Frahm J, Hemmings BA (2005). Essential role of protein kinase B gamma (PKB gamma/Akt3) in postnatal brain development but not in glucose homeostasis. Development.

