# Supplementary material for: Knockdown of Foxg1 in supporting cells increases the trans-differentiation of supporting cells into hair cells in the neonatal mouse cochlea
Source: Cell Mol Life Sci. 2019 Sep 4;77(7):1401–19. doi: 10.1007/s00018-019-03291-2 (PMC7113235; doi:10.1007/s00018-019-03291-2)
Supplement: Supplementary file 1 — Supplementary material 1 (DOCX 34 kb) [file 18_2019_3291_MOESM1_ESM.docx]

**Supplementary Information：**

**Supplementary Figure Legends：**

**Figure S1.** Breeding strategy of Sox2^CreER/+^ Foxg1^loxp/loxp^ mice. Sox2^CreER/+^ mice and Foxg1^loxp/+^ mice were crossed to get Sox2^CreER/+^ Foxg1^loxp/+^ mice. Sox2^CreER/+^ Foxg1^loxp/+^ mice were then crossed with Foxg1^loxp/+^ mice. We obtained six different genotypes of mice, and their ratios are shown in the figure.

**Figure S2.** *Foxg1* was successfully knocked down in Sox2+ SCs. (A) Tamoxifen was injected to activate Cre and conditionally knock down *Foxg1* in cochlear SCs of Sox2^CreER/+^ Foxg1^loxp/loxp^ mice. (B) Total cochlear mRNA was extracted, and the *Foxg1* mRNA expression level was detected by real-time qPCR. Four independent qPCR experiments were performed. *p < 0.05. (C-D) The Foxg1 antibody was used to detect Foxg1 protein expression in SCs (C). The HC layer is also shown in (D). Sox2 was used as the SC marker. Scale bar, 20 µm. Foxg1^loxp/loxp^ mice were used as control mice.

**Figure S3.** cKD of *Foxg1* in Sox9+ SCs also results in extra HCs. (A) Tamoxifen was injected into P1 Sox9^CreER/+^ Rosa26-tdTomato mice for 48 h to activate Cre recombinase. tdTomato was the indicator for Sox9+ cells. Scale bar, 50 µm. Note that there were no extra IHCs and OHCs in the cochleae of Sox9^CreER/+^ mice. (B) Tamoxifen was injected at P1 to conditionally knock down *Foxg1* in Sox9+ SCs. Increased IHCs (arrows) and OHCs (square brackets) were seen in the apical (Apex) and middle (Middle) turns of P7 Sox9^CreER/+^ Foxg1^loxp/loxp^ cochleae. Scale bar, 20 µm. (C) Quantification of the total IHCs, total OHCs, total SCs per 100 µm cochlea length. The n refers to the number of mice. **p < 0.01.

**Figure S4.** Body weight and ABR test of *Foxg1* cKD mice. (A) P30 Sox2^CreER/+^ Foxg1^loxp/loxp^ mice were much smaller than Foxg1^loxp/loxp^ and Sox2^CreER/+^control mice. (B) Quantification of body weight of P30 Sox2^CreER/+^ Foxg1^loxp/loxp^, Foxg1^loxp/loxp^ and Sox2^CreER/+^ mice. n = 4 mice per group. *p < 0.05. (C) ABR test of P30 Sox2^CreER/+^ Foxg1^loxp/loxp^ and Foxg1^loxp/loxp^ mice. n = 4 mice per group. *p < 0.05.

**Figure S5.** Extra IHCs were not regenerated after P7. (A) Tamoxifen was injected into P7 Sox2^CreER/+^ Foxg1^loxp/loxp^ mice and Sox2^CreER/+^ mice. Few extra IHC were found in both mice. Scale bar, 50 µm. (B) Statistical analysis of total IHCs and OHCs per 100 µm cochlea length. n.s., not significant. The n refers to the number of mice. (C) TUNEL assay of P7 Sox2^CreER/+^ Foxg1^loxp/loxp^ mice and Sox2^CreER/+^ mice cochlea. Scale bar, 20 µm.

**Figure S6.** Synapses and neuron projections of the extra IHCs. (A-B) Ctbp2 and PSD95 was used to stain synapses (dotted staining) of IHCs. Each IHC and its Ctbp2+ synapse is indicated by a dotted white circle. Extra IHCs (white arrows) and normal IHCs (yellow arrows) are shown in both confocal images. Quantification of Ctbp2+/PSD95+ synapse is shown in (B). (C) Tuj1 staining of IHC and OHC. White arrows show neuron projection to IHCs and OHCs. Scale bar, 20 µm.

**Figure S7.** tdTomato was mainly activated by Sox2-CreER in SCs. (A-B) Tamoxifen was injected at P1 in to Sox2^CreRE/+^Rosa26-tdTomato mice and the mice were sacrificed at P3. The yellow bracket in ① and the white bracket in ② show the region in which some of the HCs were labeled by tdTomato. The other tdTomato+ HCs are indicated by arrowheads. Scale bar, 20 µm. (C-D) Tamoxifen was injected at P3 into Sox2^CreRE/+^Rosa26-tdTomato mice, and the mice were sacrificed at P5. The white bracket shows that some of the HCs in the apex tip region were labeled by tdTomato. The other tdTomato+ HCs are indicated by arrow heads. Scale bar, 20 µm. (E) Tamoxifen was injected at P1 in to Lgr5-EGFP^CreRE/+^Rosa26-tdTomato mice and the mice was sacrificed at P3. Scale bar, 20 µm.

**Supplementary Table 1**

Primers for real-time qPCR detection and relative quantification of gene expression in mouse

| Gene name | primer | Real-time qPCR primer sequence (5'–3') |
| --- | --- | --- |
| *Gapdh* | Forward | AGGTCGGTGTGAACGGATTTG |
|  | Reverse | TGTAGACCATGTAGTTGAGGTCA |
| *Foxg1* | Forward | AGCGACGACGTGTTCATCG |
|  | Reverse | CCCGTTGTAACTCAAAGTGCTG |
| *Atoh1* | Forward | GAGTGGGCTGAGGTAAAAGAGT |
|  | Reverse | GGTCGGTGCTATCCAGGAG |
| *Pou4f3* | Forward | ATGCGCCGAGTTTGTCTCC |
|  | Reverse | GGGCTTGAACGGATGATTCTTG |
| *Gfi1* | Forward | AGAAGGCGCACAGCTATCAC |
|  | Reverse | GGCTCCATTTTCGACTCGC |
| *Neurog1* | Forward | CCAGCGACACTGAGTCCTG |
|  | Reverse | CGGGCCATAGGTGAAGTCTT |
| *Cdkn1a* | Forward | CCTGGTGATGTCCGACCTG |
|  | Reverse | CCATGAGCGCATCGCAATC |
| *Cdkn1c* | Forward | CGAGGAGCAGGACGAGAATC |
|  | Reverse | GAAGAAGTCGTTCGCATTGGC |
| *Cdkn2a* | Forward | CGCAGGTTCTTGGTCACTGT |
|  | Reverse | TGTTCACGAAAGCCAGAGCG |
| *Gadd45g* | Forward | GGGAAAGCACTGCACGAACT |
|  | Reverse | AGCACGCAAAAGGTCACATTG |
| *Tfdp1* | Forward | TTGAAGCCAACGGAGAACTAAAG |
|  | Reverse | TGGACTGTCCGAAGGTTTTTG |
| *Wee1* | Forward | GTCGCCCGTCAAATCACCTT |
|  | Reverse | GAGCCGGAATCAATAACTCGC |
| *Cdk2* | Forward | CCTGCTTATCAATGCAGAGGG |
|  | Reverse | GTGCTGGGTACACACTAGGTG |
| *Cdk4* | Forward | ATGGCTGCCACTCGATATGAA |
|  | Reverse | TCCTCCATTAGGAACTCTCACAC |
| *Cdk6* | Forward | GGCGTACCCACAGAAACCATA |
|  | Reverse | AGGTAAGGGCCATCTGAAAACT |
| *Cdc25a* | Forward | ACAGCAGTCTACAGAGAATGGG |
|  | Reverse | GATGAGGTGAAAGGTGTCTTGG |
| *Cdc25b* | Forward | TCCGATCCTTACCAGTGAGG |
|  | Reverse | GGTCTCTGGAAGCGCACATT |
| *Cdc25c* | Forward | ATGTCTACAGGACCTATCCCAC |
|  | Reverse | ACCTAAAACTGGGTGCTGAAAC |
| *Ccnd1* | Forward | GCGTACCCTGACACCAATCTC |
|  | Reverse | CTCCTCTTCGCACTTCTGCTC |
| *Axin1* | Forward | CTCCAAGCAGAGGACAAAATCA |
|  | Reverse | GGATGGGTTCCCCACAGAAATA |
| *GSK3β* | Forward | TGGCAGCAAGGTAACCACAG |
|  | Reverse | CGGTTCTTAAATCGCTTGTCCTG |
| *Ctnnb1* | Forward | ATGGAGCCGGACAGAAAAGC |
|  | Reverse | CTTGCCACTCAGGGAAGGA |
| *Lgr5* | Forward | TCTTCACCTCCTACCTGGACCT |
|  | Reverse | GGCGTAGTCTGCTATGTGGTGT |
| *Axin2* | Forward | TGACTCTCCTTCCAGATCCCA |
|  | Reverse | TGCCCACACTAGGCTGACA |
| *Myc* | Forward | ATGCCCCTCAACGTGAACTTC |
|  | Reverse | CGCAACATAGGATGGAGAGCA |
| *Dkk2* | Forward | CTGATGCGGGTCAAGGATTCA |
|  | Reverse | CTCCCCTCCTAGAGAGGACTT |
| *Notch1* | Forward | CCGTGTAAGAATGCTGGAACG |
|  | Reverse | AGCGACAGATGTATGAAGACTCA |
| *Notch2* | Forward | ATGTGGACGAGTGTCTGTTGC |
|  | Reverse | GGAAGCATAGGCACAGTCATC |
| *Notch3* | Forward | TGCCAGAGTTCAGTGGTGG |
|  | Reverse | CACAGGCAAATCGGCCATC |
| *Hes1* | Forward | CCAGCCAGTGTCAACACGA |
|  | Reverse | AATGCCGGGAGCTATCTTTCT |
| *Hes5* | Forward | AGTCCCAAGGAGAAAAACCGA |
|  | Reverse | GCTGTGTTTCAGGTAGCTGAC |
| *Jag1* | Forward | CCTCGGGTCAGTTTGAGCTG |
|  | Reverse | CCTTGAGGCACACTTTGAAGTA |
| *Jag2* | Forward | CAATGACACCACTCCAGATGAG |
|  | Reverse | GGCCAAAGAAGTCGTTGCG |
| *Hey1* | Forward | GCGCGGACGAGAATGGAAA |
|  | Reverse | TCAGGTGATCCACAGTCATCTG |
| *Hey2* | Forward | AAGCGCCCTTGTGAGGAAAC |
|  | Reverse | GGTAGTTGTCGGTGAATTGGAC |
| *Dll1* | Forward | CAGGACCTTCTTTCGCGTATG |
|  | Reverse | AAGGGGAATCGGATGGGGTT |
| *Dll4* | Forward | TTCCAGGCAACCTTCTCCGA |
|  | Reverse | ACTGCCGCTATTCTTGTCCC |
| *Tle1* | Forward | CCAGTACCTCTCACGCCTCA |
|  | Reverse | GCCCACTCAGAGCACTAGAC |
| *Tle2* | Forward | TGGCTGCCGTAAAGGAAGAC |
|  | Reverse | CTCACTGTCATAAGGCCCTGA |
| *Tgfbr1* | Forward | TCCCAACTACAGGACCTTTTTCA |
|  | Reverse | GCAGTGGTAAACCTGATCCAGA |
| *Tgfbr2* | Forward | CCGCTGCATATCGTCCTGTG |
|  | Reverse | AGTGGATGGATGGTCCTATTACA |
| *Smad2* | Forward | ATGTCGTCCATCTTGCCATTC |
|  | Reverse | AACCGTCCTGTTTTCTTTAGCTT |
| *Smad3* | Forward | CACGCAGAACGTGAACACC |
|  | Reverse | GGCAGTAGATAACGTGAGGGA |
| *Smad4* | Forward | ACACCAACAAGTAACGATGCC |
|  | Reverse | GCAAAGGTTTCACTTTCCCCA |
| *Smad7* | Forward | GGCCGGATCTCAGGCATTC |
|  | Reverse | TTGGGTATCTGGAGTAAGGAGG |
| *Bmpr1a* | Forward | AACAGCGATGAATGTCTTCGAG |
|  | Reverse | GTCTGGAGGCTGGATTATGGG |
| *Bmpr1b* | Forward | CCCTCGGCCCAAGATCCTA |
|  | Reverse | CAACAGGCATTCCAGAGTCATC |
| *Bmpr2* | Forward | TTGGGATAGGTGAGAGTCGAAT |
|  | Reverse | TGTTTCACAAGATTGATGTCCCC |
| *Ltbp1* | Forward | CCAGTCCCAAGTCTCTTACCA |
|  | Reverse | CTGGAAGCATCGGCCAAGT |
| *Sox2* | Forward | ATGAACGGCTGGAGCAACGGCA |
|  | Reverse | TCACATGTGCGACAGGGGCAGT |

**Supplementary Table 2**

HC and SC number quantification of P7 Sox2^CreER/+^ Foxg1^loxp/loxp^ mice

|  | Sox2^CreER/+^ Foxg1^loxp/loxp^ | Sox2^CreER/+^ | Foxg1^loxp/loxp^ |
| --- | --- | --- | --- |
| Extra IHCs per turn (Apex) | 27.67 ± 3.85 | 13.6 ± 1.99 | 5.75 ± 0.75 |
| Extra IHCs per turn (Middle) | 23.5 ± 3.34 | 8.0 ± 1.71 | 0.75 ± 0.75 |
| Extra IHCs per turn (Base) | 7.17 ± 1.66 | 8.2 ± 1.83 | 0.0 ± 0.0 |
| Total IHCs per 100 µm (Apex) | 14.41 ± 0.29 | 11.93 ± 0.25 |  |
| Total IHCs per 100 µm (Middle) | 14.91 ± 0.2 | 12.92 ± 0.31 |  |
| Total IHCs per 100 µm (Base) | 13.09 ± 0.36 | 12.61 ± 0.36 |  |
| Total OHCs per 100 µm (Apex) | 43.06 ± 1.41 | 39.43 ± 1.09 |  |
| Total OHCs per 100 µm (Middle) | 39.50 ± 0.54 | 40.73 ± 0.7 |  |
| Total OHCs per 100 µm (Base) | 37.47 ± 0.92 | 38.75 ± 0.72 |  |
| Total SCs per 100 µm (Apex) | 68.59 ± 1.72 | 72.92 ± 0.73 |  |
| Total SCs per 100 µm (Middle) | 70.0 ± 0.94 | 73.65 ± 0.86 |  |
| Total SCs per 100 µm (Base) | 66.13 ± 1.09 | 68.96 ± 1.37 |  |
| Total DCs per 100 µm (Apex) | 38.0 ± 1.47 | 40.31 ± 0.64 |  |
| Total DCs per 100 µm (Middle) | 37.87 ± 0.45 | 40.47 ± 0.81 |  |
| Total DCs per 100 µm (Base) | 35.56 ± 0.76 | 36.82 ± 0.87 |  |
| Total OPCs per 100 µm (Apex) | 12.88 ± 0.13 | 13.65 ± 0.17 |  |
| Total OPCs per 100 µm (Middle) | 13.13 ± 0.24 | 14.06 ± 0.12 |  |
| Total OPCs per 100 µm (Base) | 12.44 ± 0.23 | 13.13 ± 0.43 |  |
| Total IPCs per 100 µm (Apex) | 17.63 ± 0.44 | 18.96 ± 0.19 |  |
| Total IPCs per 100 µm (Middle) | 19.0 ± 0.34 | 19.12 ± 0.19 |  |
| Total IPCs per 100 µm (Base) | 18.13 ± 0.3 | 19.01 ± 0.27 |  |

**Supplementary Table 3**

HC and SC number quantification of P7 Lgr5-EGFP^CreER/+^ Foxg1^loxp/loxp^ mice

|  | Lgr5-EGFP^CreER/+^ Foxg1^loxp/loxp^ | Lgr5-EGFP^CreER/+^ | Foxg1^loxp/loxp^ |
| --- | --- | --- | --- |
| Extra IHCs per turn (Apex) | 14.4 ± 1.21 | 5.6 ± 0.93 | 4.0 ± 1.08 |
| Extra IHCs per turn (Middle) | 8.0 ± 2.0 | 2.8 ± 0.86 | 1.0 ± 0.41 |
| Extra IHCs per turn (Base) | 1.4 ± 0.87 | 1.6 ±0.51 | 0.5 ± 0.29 |
| Total IHCs per 100 µm (Apex) | 14.53 ± 0.39 | 11.67 ± 0.31 |  |
| Total IHCs per 100 µm (Middle) | 14.53 ± 0.30 | 13.23 ± 0.25 |  |
| Total IHCs per 100 µm (Base) | 14.06 ± 1.07 | 12.71 ± 0.26 |  |
| Total OHCs per 100 µm (Apex) | 40.0 ± 1.22 | 37.19 ± 0.68 |  |
| Total OHCs per 100 µm (Middle) | 40.31 ± 1.62 | 40.83 ± 0.42 |  |
| Total OHCs per 100 µm (Base) | 38.44 ± 0.54 | 39.06 ± 0.53 |  |
| Total SCs per 100 µm (Apex) | 70.78 ± 2.76 | 71.46 ± 1.55 |  |
| Total SCs per 100 µm (Middle) | 73.91 ± 1.41 | 74.48 ± 0.59 |  |
| Total SCs per 100 µm (Base) | 70.94 ± 0.74 | 70.52 ± 1.13 |  |
| Total DCs per 100 µm (Apex) | 40.94 ± 1.16 | 40.42 ± 1.82 |  |
| Total DCs per 100 µm (Middle) | 41.41 ± 1.03 | 40.52 ± 0.47 |  |
| Total DCs per 100 µm (Base) | 38.44 ± 0.4 | 38.13 ± 0.98 |  |
| Total OPCs per 100 µm (Apex) | 12.66 ± 0.16 | 12.92 ± 0.13 |  |
| Total OPCs per 100 µm (Middle) | 13.13 ± 0.26 | 13.85 ± 0.19 |  |
| Total OPCs per 100 µm (Base) | 12.66 ± 0.3 | 13.44 ± 0.27 |  |
| Total IPCs per 100 µm (Apex) | 17.19 ± 1.56 | 18.13 ± 0.32 |  |
| Total IPCs per 100 µm (Middle) | 19.38 ± 0.44 | 20.1 ± 0.38 |  |
| Total IPCs per 100 µm (Base) | 19.84 ± 0.47 | 19.17 ± 0.21 |  |

**Supplementary Table 4**

HC and SC number quantification of P7 Sox9^CreER/+^ Foxg1^loxp/loxp^ mice

|  | Sox9^CreER/+^ Foxg1^loxp/loxp^ | Sox9^CreER/+^ |
| --- | --- | --- |
| Total IHCs per 100 µm (Apex) | 14.5 ± 0.46 | 11.67 ± 0.55 |
| Total IHCs per 100 µm (Middle) | 12.63 ± 0.13 | 12.71 ± 0.21 |
| Total IHCs per 100 µm (Base) | 11.25 ± 0.79 | 11.88 ± 0.63 |
| Total OHCs per 100 µm (Apex) | 47.0 ± 1.11 | 39.58 ± 1.78 |
| Total OHCs per 100 µm (Middle) | 41.38 ± 1.0 | 40.83 ± 1.16 |
| Total OHCs per 100 µm (Base) | 38.38 ± 1.06 | 37.92 ± 0.91 |
| Total SCs per 100 µm (Apex) | 71.13 ± 1.02 | 71.67 ± 2.66 |
| Total SCs per 100 µm (Middle) | 72.25 ± 0.81 | 72.92 ± 1.71 |
| Total SCs per 100 µm (Base) | 68.63 ± 2.01 | 68.96 ± 1.82 |
| Total DCs per 100 µm (Apex) | 41.5 ± 0.67 | 41.04 ± 1.71 |
| Total DCs per 100 µm (Middle) | 42.38 ± 0.54 | 40.42 ± 1.1 |
| Total DCs per 100 µm (Base) | 39.0 ± 1.8182 | 37.92 ± 0.91 |
| Total OPCs per 100 µm (Apex) | 12.63 ± 0.23 | 13.13 ± 0.63 |
| Total OPCs per 100 µm (Middle) | 12.75 ± 0.15 | 13.75 ± 0.36 |
| Total OPCs per 100 µm (Base) | 12.38 ± 0.23 | 12.92 ± 0.55 |
| Total IPCs per 100 µm (Apex) | 17.25 ± 0.15 | 17.5 ± 0.36 |
| Total IPCs per 100 µm (Middle) | 17.13 ± 0.32 | 18.75 ± 0.36 |
| Total IPCs per 100 µm (Base) | 17.38 ± 0.13 | 18.13 ± 0.36 |
