# Supplementary figures and images for: Knockdown of Foxg1 in supporting cells increases the trans-differentiation of supporting cells into hair cells in the neonatal mouse cochlea
Source: Cell Mol Life Sci. 2019 Sep 4;77(7):1401–19. doi: 10.1007/s00018-019-03291-2 (PMC7113235; doi:10.1007/s00018-019-03291-2)

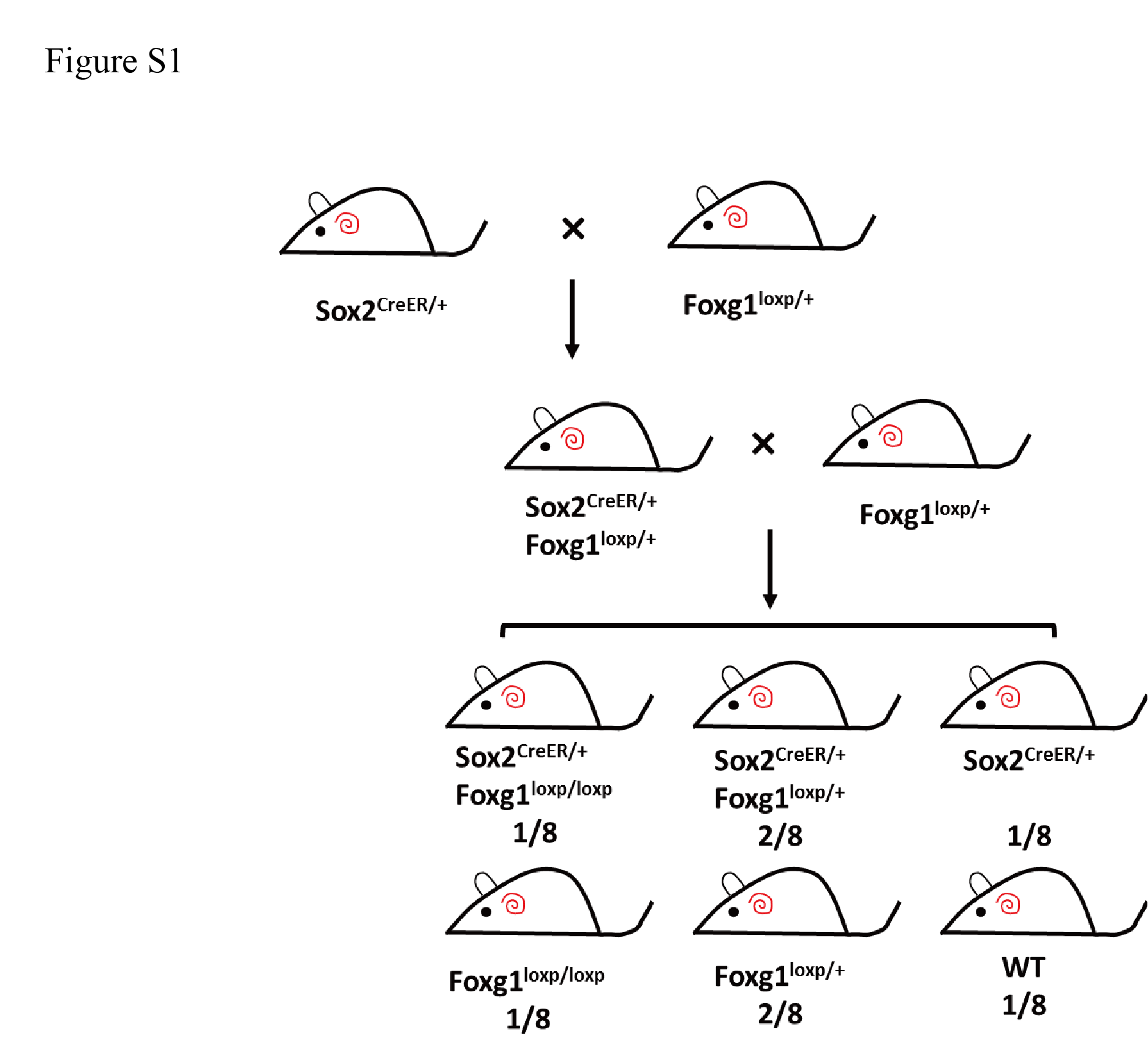

Supplement: Supplementary file 2 — Supplementary material 2 (TIFF 386 kb) [file 18_2019_3291_MOESM2_ESM.tif]

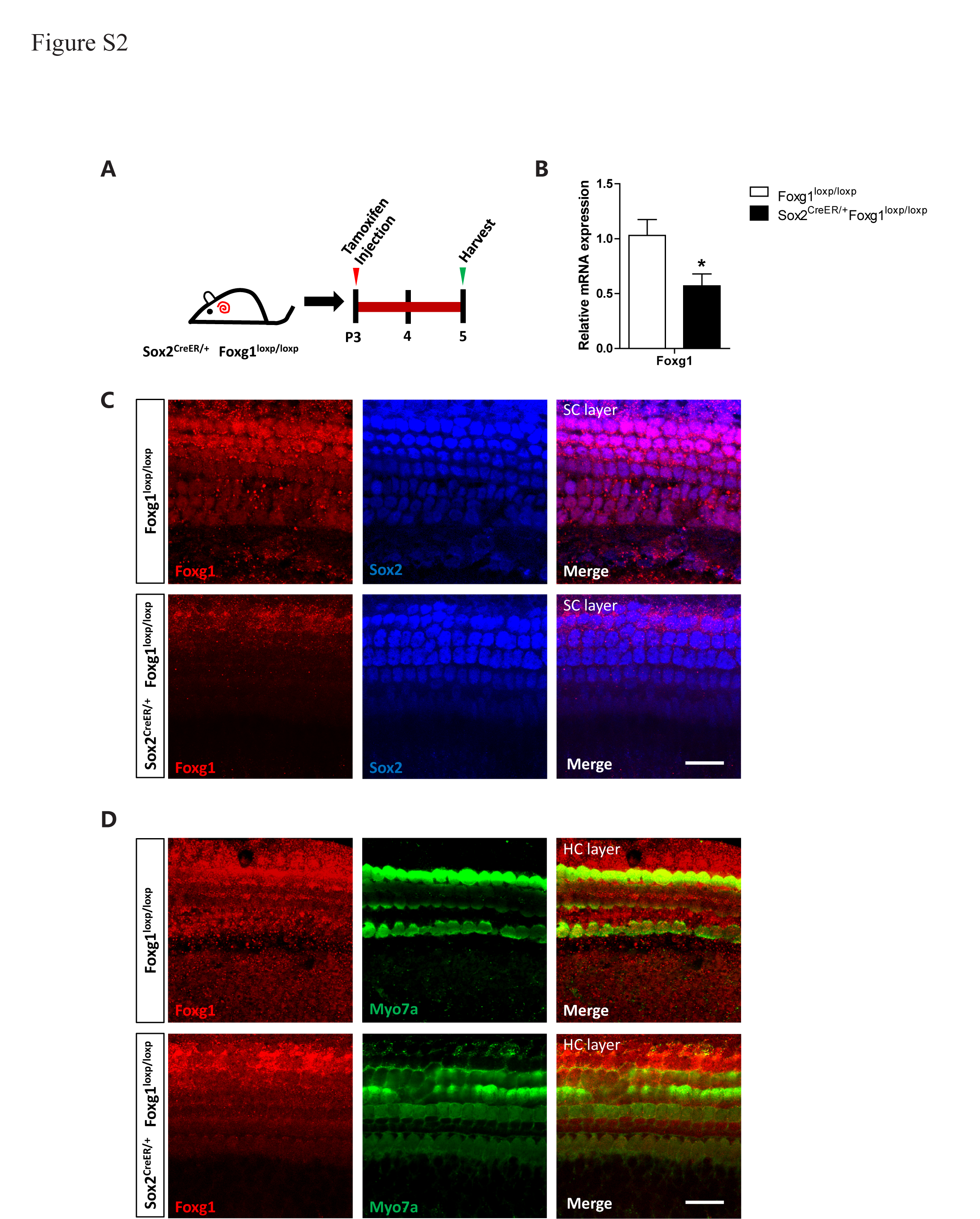

Supplement: Supplementary file 3 — Supplementary material 3 (TIFF 4192 kb) [file 18_2019_3291_MOESM3_ESM.tif]

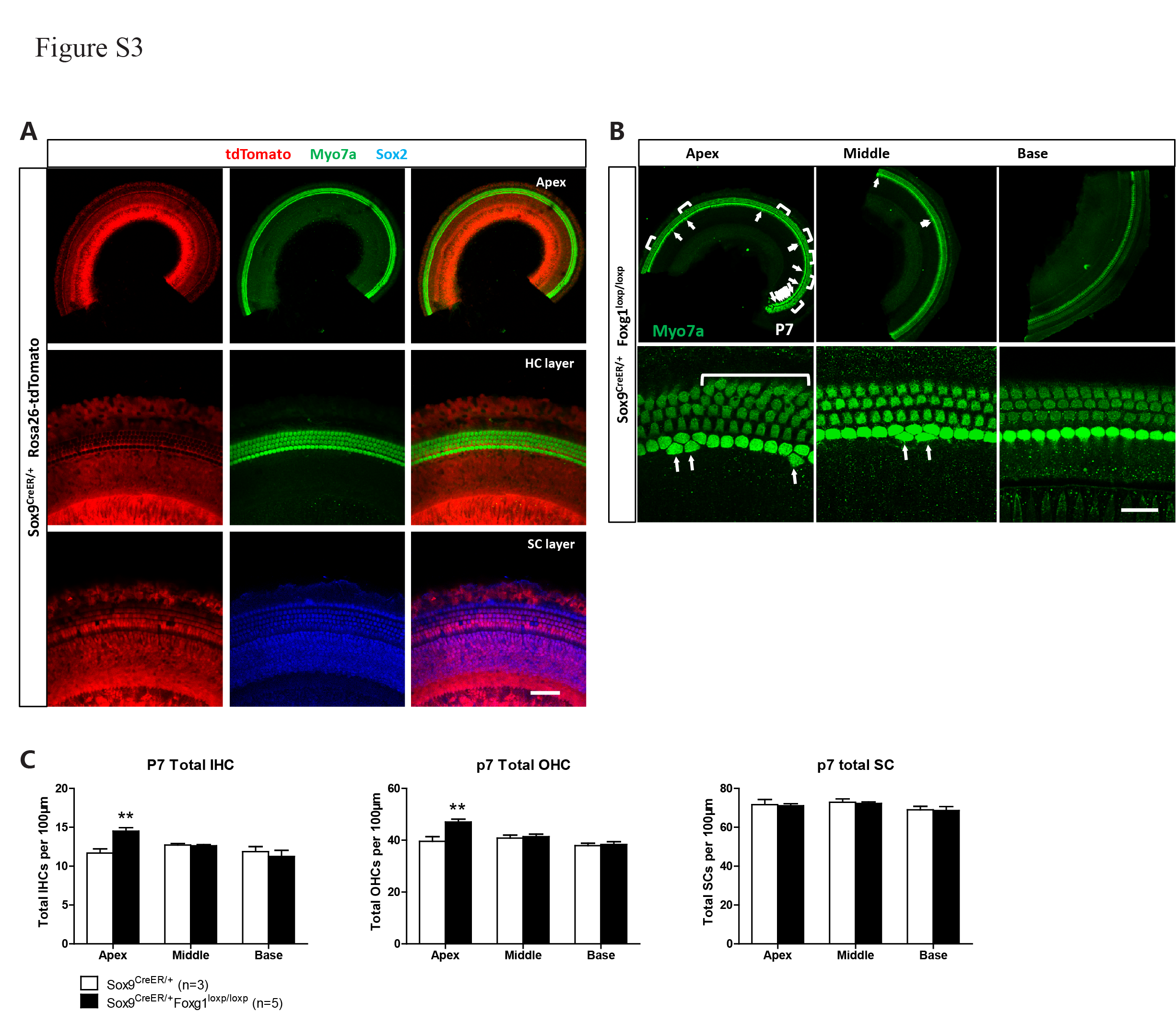

Supplement: Supplementary file 4 — Supplementary material 4 (TIFF 2720 kb) [file 18_2019_3291_MOESM4_ESM.tif]

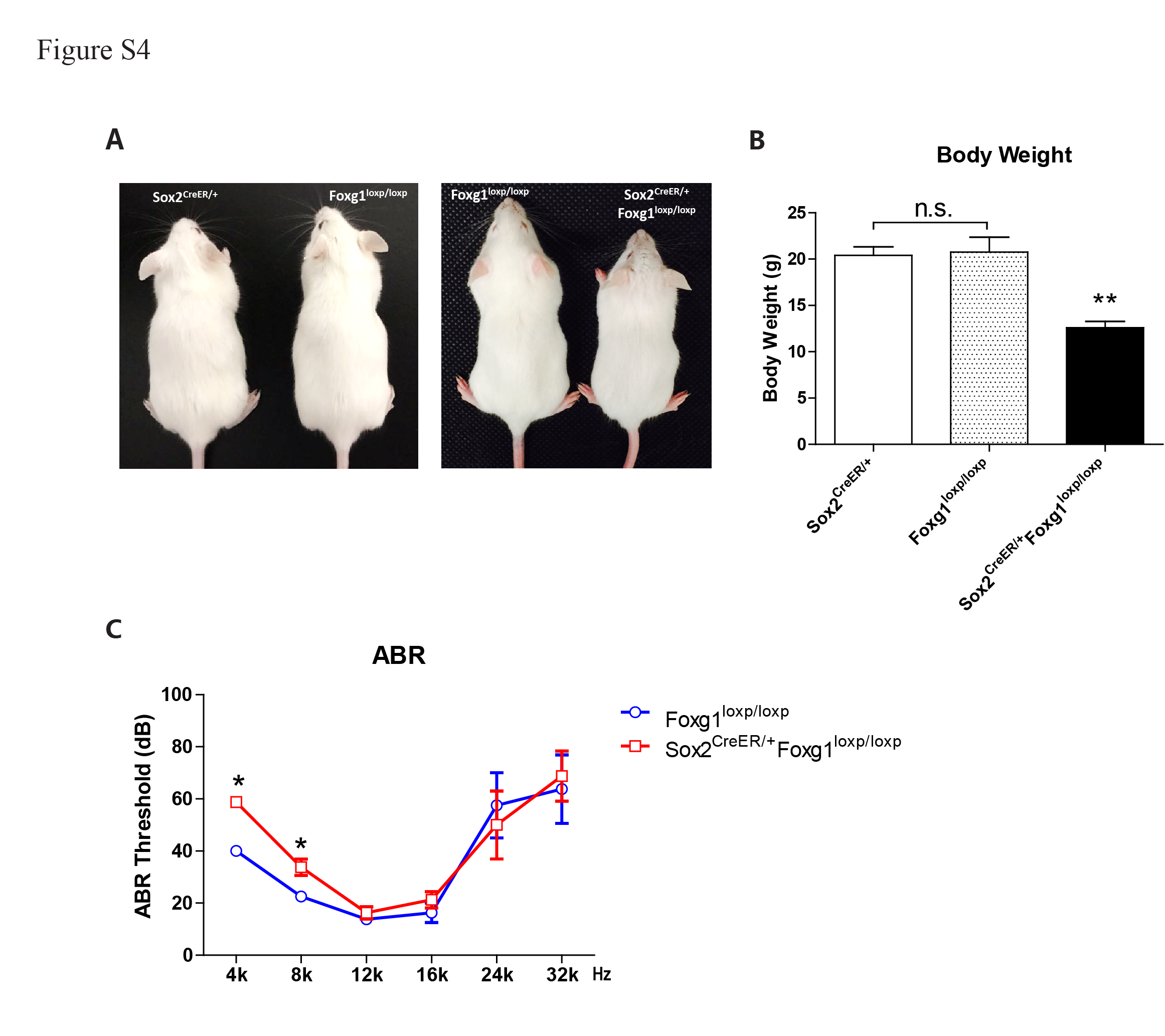

Supplement: Supplementary file 5 — Supplementary material 5 (TIFF 1806 kb) [file 18_2019_3291_MOESM5_ESM.tif]

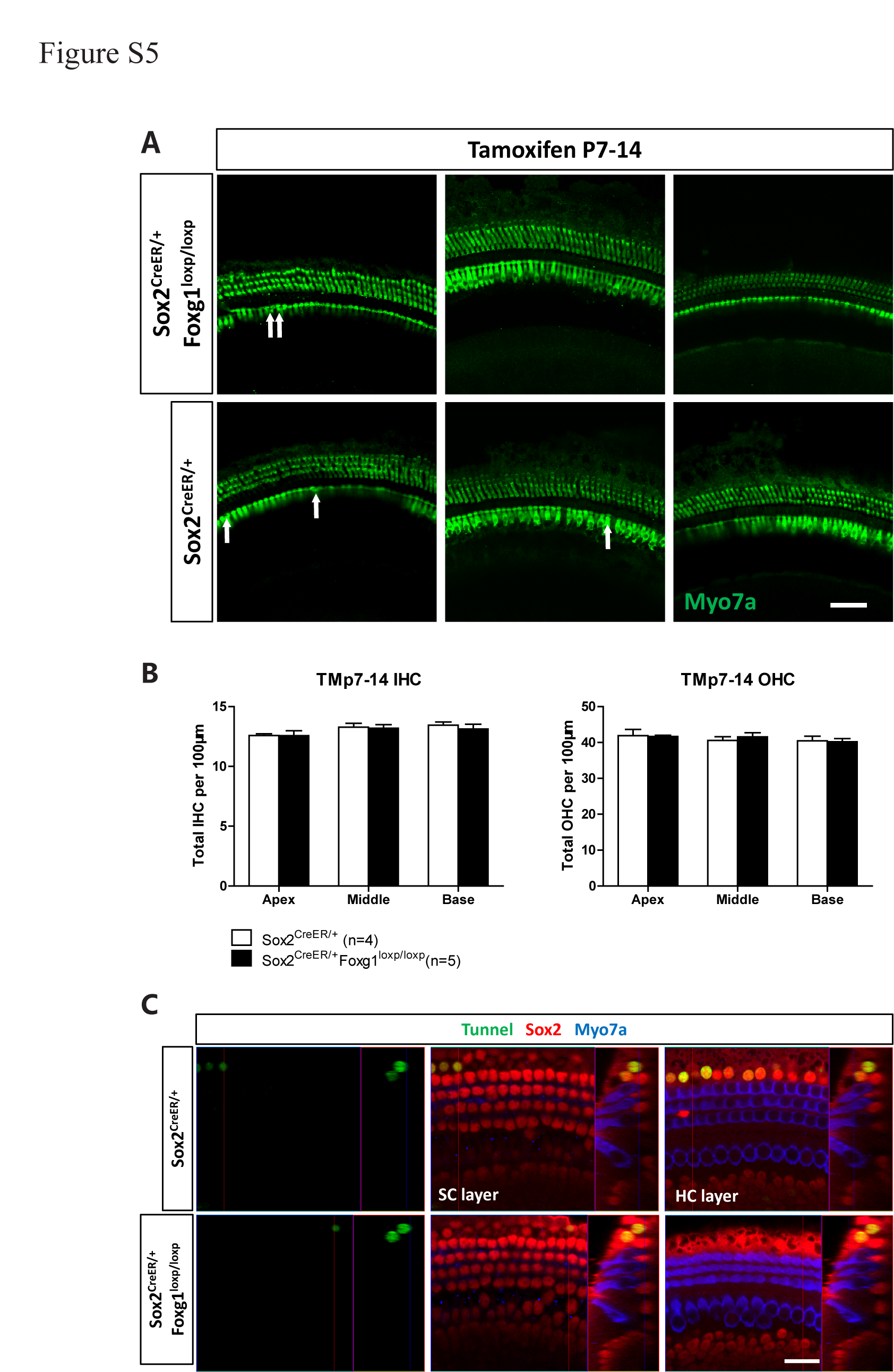

Supplement: Supplementary file 6 — Supplementary material 6 (TIFF 2362 kb) [file 18_2019_3291_MOESM6_ESM.tif]

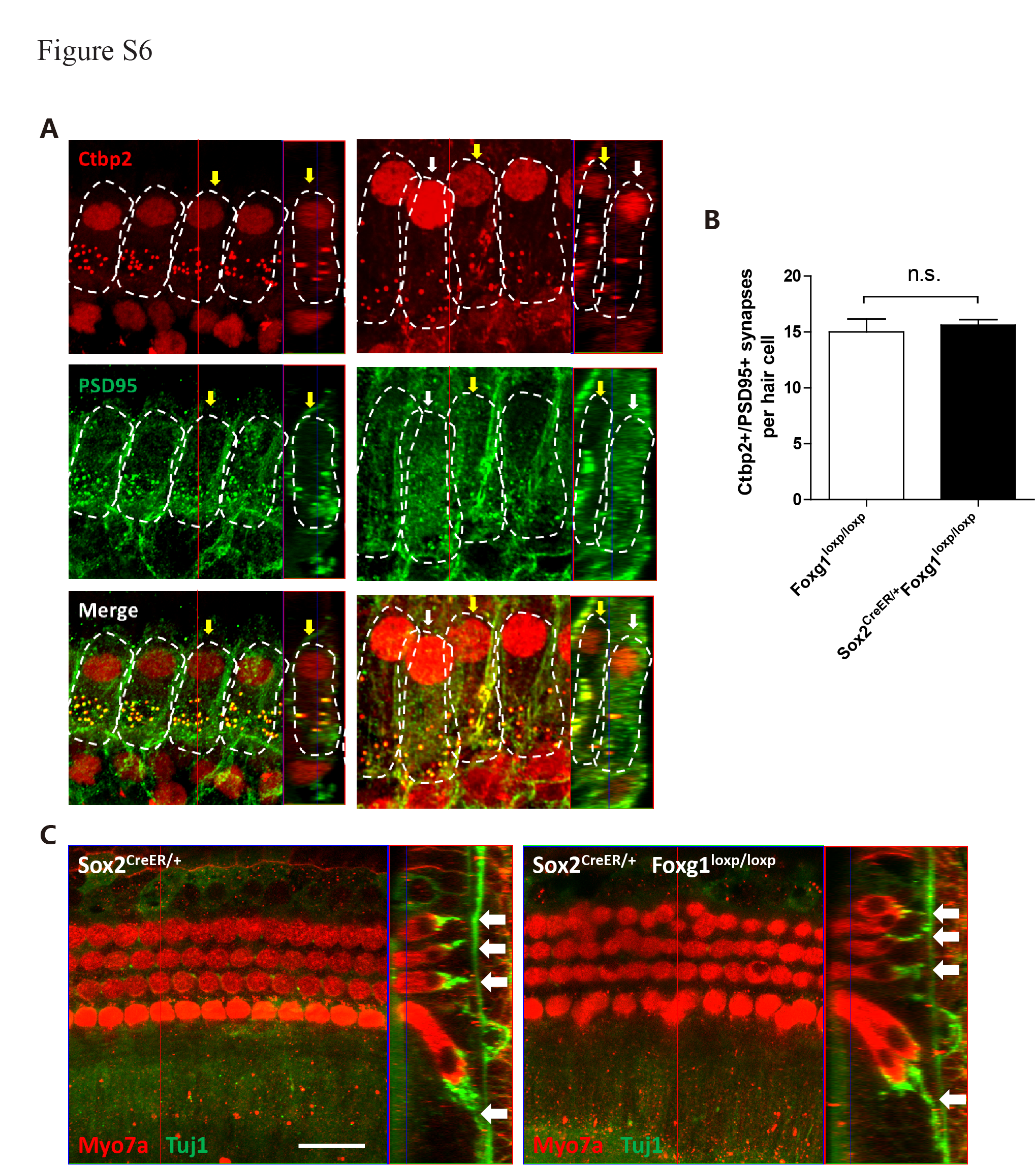

Supplement: Supplementary file 7 — Supplementary material 7 (TIFF 5324 kb) [file 18_2019_3291_MOESM7_ESM.tif]

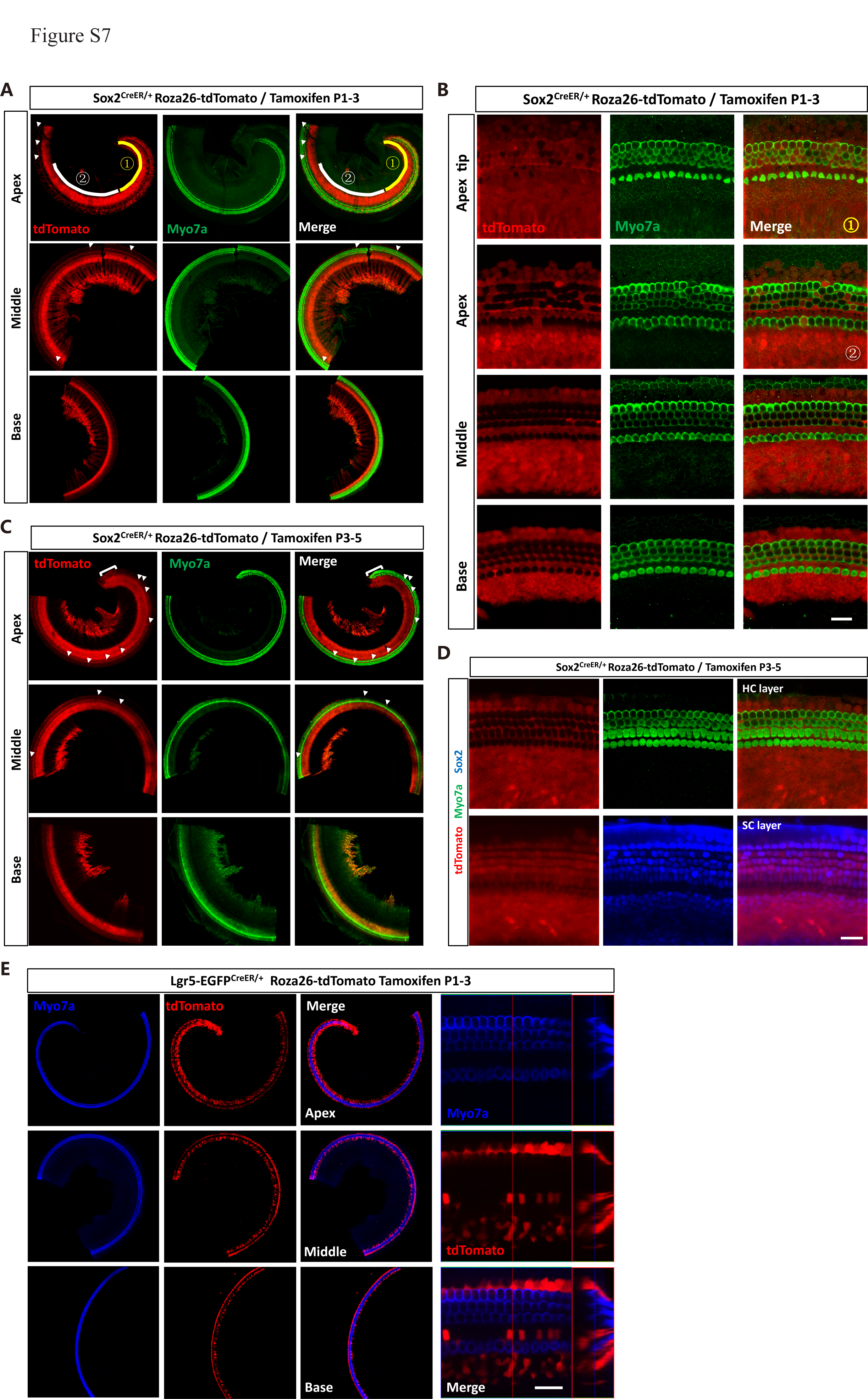

Supplement: Supplementary file 8 — Supplementary material 8 (TIFF 7519 kb) [file 18_2019_3291_MOESM8_ESM.tif]
